# The R2R3 MYB transcription factor *PavMYB10.1* involves in anthocyanin biosynthesis and determines fruit skin colour in sweet cherry (*Prunus avium* L.)

**DOI:** 10.1111/pbi.12568

**Published:** 2016-05-19

**Authors:** Wanmei Jin, Hua Wang, Maofu Li, Jing Wang, Yuan Yang, Xiaoming Zhang, Guohua Yan, Hong Zhang, Jiashen Liu, Kaichun Zhang

**Affiliations:** ^1^ Institute of Forestry and Pomology Beijing Academy of Agriculture and Forestry Sciences Beijing China; ^2^ Key Laboratory of Biology and Genetic Improvement of Horticultural Crops (North China) Ministry of Agriculture Beijing China; ^3^ Beijing Engineering Research Center for Deciduous Fruit Trees Beijing China

**Keywords:** *Prunus avium*, fruit skin colour, MYB regulation, alleles, colour traits, inheritance

## Abstract

Sweet cherry is a diploid tree species and its fruit skin has rich colours from yellow to blush to dark red. The colour is closely related to anthocyanin biosynthesis and is mainly regulated at the transcriptional level by transcription factors that regulate the expression of multiple structural genes. However, the genetic and molecular bases of how these genes ultimately determine the fruit skin colour traits remain poorly understood. Here, our genetic and molecular evidences identified the R2R3 MYB transcription factor *PavMYB10.1* that is involved in anthocyanin biosynthesis pathway and determines fruit skin colour in sweet cherry. Interestingly, we identified three functional alleles of the gene causally leading to the different colours at mature stage. Meanwhile, our experimental results of yeast two‐hybrid assays and chromatin immunoprecipitation assays revealed that PavMYB10.1 might interact with proteins PavbHLH and PavWD40, and bind to the promoter regions of the anthocyanin biosynthesis genes *PavANS* and *PavUFGT*; these findings provided to a certain extent mechanistic insight into the gene's functions. Additionally, genetic and molecular evidences confirmed that *PavMYB10.1* is a reliable DNA molecular marker to select fruit skin colour in sweet cherry.

## Introduction

Anthocyanins, which are derived from the phenylpropanoid pathway, belong to the flavonoids class of secondary metabolites. They are responsible for the purple, red and blue coloration of leaves, stems, flowers and fruits of many plants (Cominelli *et al*., 2008; Das *et al*., 2012; Espley *et al*., 2007; Hatlestad, 2012; Hatlestad *et al*., 2015; Pilu *et al*., 2003). Anthocyanins are a marker of ripeness in fruit, and their accumulation in fruit allows consumers to differentiate among cultivars. In particular, the red colour of fruit skin is an important determinant of consumer preference and marketability (Allan *et al*., 2008; Jaakola, 2013; Jimenez‐Garcia *et al*., 2013). Anthocyanin biosynthesis is mainly regulated at the transcriptional level by transcription factors (TFs) that control the expression of structural genes. The TFs that control anthocyanin biosynthesis include MYB TFs, basic helix‐loop‐helix (bHLH) TFs and the Trp‐Asp forty amino acid repeat (WD40) proteins (Allan *et al*., 2008; Jaakola, 2013; Jimenez‐Garcia *et al*., 2013; Petroni and Tonelli, 2011; Xu *et al*., 2015). MYB TFs play a key role, especially R2R3‐MYB class. R2R3‐MYB TFs consist of an N‐terminal conserved MYB domain and a C terminal variable activation or repression domain (Dubos *et al*., 2010). In fruits, anthocyanin biosynthesis is controlled by a distinct clade of R2R3 MYB TFs (Allan *et al*., 2008). In apple (*Malus *× *domestica* Borkh.), the transcript levels of R2R3‐MYB *MdMYB10* alleles were shown to be correlated with anthocyanin accumulation and were higher in red‐ than in green‐fruited cultivars (Ban *et al*., 2007; Takos *et al*., 2006; Telias *et al*., 2011). In grape (*Vitis vinifera*), the skin colour phenotype is controlled by a single genetic locus. Grape cultivars can be classified as either red or white based on the presence or absence of anthocyanins in the berry skins. A retrotransposon‐induced mutation in *VvmybA1*, a homologue of *VvmybA1‐1*, was shown to be associated with the loss of pigmentation in white cultivars of *V. vinifera* (Cutanda‐Perez *et al*., 2009; Kobayashi *et al*., 2004; Walker *et al*., 2007). The regulatory gene *VvMYBA1*, which activates anthocyanin biosynthesis, was not transcribed in white berries owing to the presence of a retrotransposon in its promoter (named *VvMYBA2*). The white berry allele *VvMYBA2* is inactivated by two nonconservative mutations: one leads to an amino acid substitution and the other to a frameshift resulting in a truncated protein (Cutanda‐Perez *et al*., 2009). Many reports show that the R2R3‐MYB TFs interact with common bHLH and WDR factors form a MYB–bHLH–WD40 (MBW) transcriptional activator complex to regulate the anthocyanin biosynthesis. The MBW activator complex regulates anthocyanin biosynthesis by directly activating the expression of the anthocyanin biosynthesis genes (Albert *et al*., 2011, 2014; D'Amelia *et al*., 2014; Gonzalez *et al*., 2008; Lowry *et al*., 2012; Matsui *et al*., 2008; Schaart *et al*., 2013; Starkevič *et al*., 2015; Wang *et al*., 2015a,b).

Sweet cherry (*Prunus avium* L.) is a diploid tree species that is an economically important horticultural crop worldwide. The duration from full bloom to fruit maturity is only 1.5–2 months. Sweet cherry fruits have rich skin colours ranging from yellow to blush to dark red. The difference between the red and yellow fruits is the presence or absence of anthocyanins. In sweet cherry, fruit skin colours vary widely because of differences in pigment profiles. Anthocyanins are responsible for the red colour of sweet cherry fruit skins. Different research groups have independently identified R2R3 MYB TFs responsible for anthocyanin accumulation in sweet cherry fruit. These TFs have been named *PavMYB1*,* PavMYB10* and *PavMYBA* (Lin‐Wang *et al*., 2010; Shen *et al*., 2014; Sooriyapathirana *et al*., 2010; Starkevič *et al*., 2015). The R2R3‐MYB TF PavMYBA from red‐coloured sweet cherry was shown to activate the promoters of *PavDFR*,* PavANS* and *PavUFGT*. The immature seeds of transgenic *Arabidopsis* plants overexpressing *PavMYBA* exhibited ectopic pigmentation. *PavMYBA* was shown to play an important role in ABA‐regulated anthocyanin biosynthesis (Shen *et al*., 2014). *PavMYB10* was mapped on linkage 3 (LG 3) utilizing a QTL approach, which was the major molecular determinant of red coloration in sweet cherry (Sooriyapathirana *et al*., 2010). Several *MYB10* genes were isolated and analysed from different cultivars of sweet cherry. By homology to the related peach genes, the shorter gene was named *PaMYB10.1*, and the longer was named *PaMYB10.2* (Starkevič *et al*., 2015). The role of the homologues and orthologues of these genes as regulators of anthocyanin biosynthesis has been shown previously in *Prunus* species. However, the relationships between these genes and the fruit colour trait remain poorly understand.

In this study, we explored the R2R3‐MYB TFs regulation of anthocyanin biosynthesis in three differently coloured sweet cherry varieties: the dark‐red variety ‘Lapins’, the blush variety ‘Rainier’ and the yellow variety ‘Big Dragon’ (Figure S1). We demonstrate that *PavMYB10.1* plays a key role in regulating anthocyanin biosynthesis in sweet cherry. Different alleles confer the different fruit colours of sweet cherry varieties.

## Results

### Anthocyanin accumulation during sweet cherry fruit development

**Figure 1:**
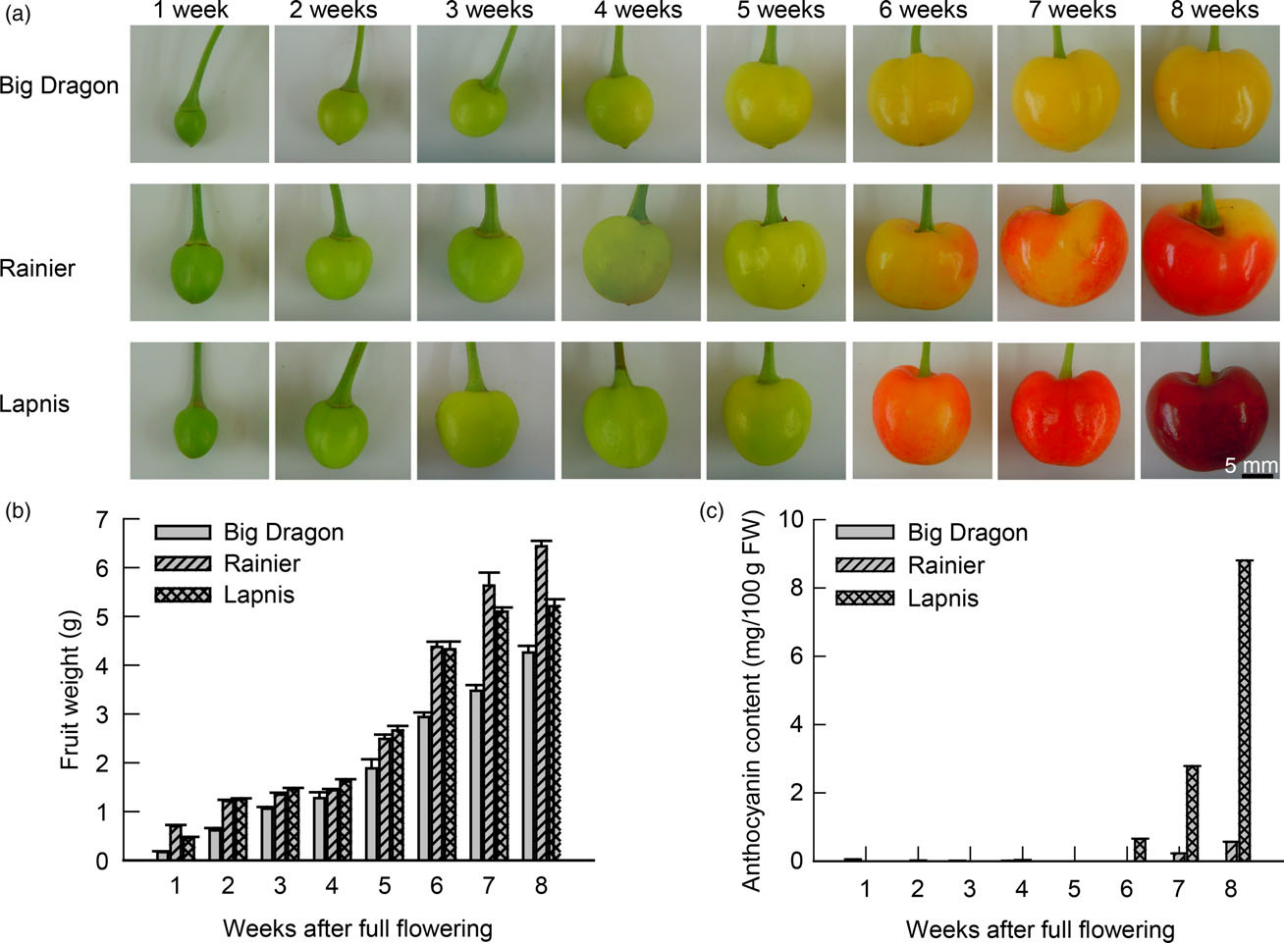
Fruit development and anthocyanin accumulation during sweet cherry fruit development. (a) Process of fruit development in sweet cherry varieties ‘Big Dragon’, ‘Rainier’ and ‘Lapins’. (b) Fruit weight of three sweet cherry varieties during fruit development. (c) Anthocyanin contents in fruit of three sweet cherry varieties during fruit development.

Based on the fruit skin colour of ‘Lapins’, eight stages of fruit development were defined for the three cultivars, from 1 to 8 weeks (Figure 1a). The fruit weight increased continuously from 1 to 8 weeks (Figure 1b), whereas the anthocyanin content increased rapidly from 6 to 8 weeks. The anthocyanin content of ‘Lapins’ increased rapidly after 6 weeks and anthocyanin accumulation was visible at 8 weeks. The anthocyanin content of ‘Rainier’ increased slowly from 6 to 8 weeks. No anthocyanins were detected in ‘Big Dragon’ (Figure 1c). These results revealed that the difference between the yellow and red fruit was the presence or absence of anthocyanins and the key stages in colour development were 6–8 weeks after full bloom.

### Mapping, identification and analysis of the R2R3‐MYB TF genes

**Figure 2:**
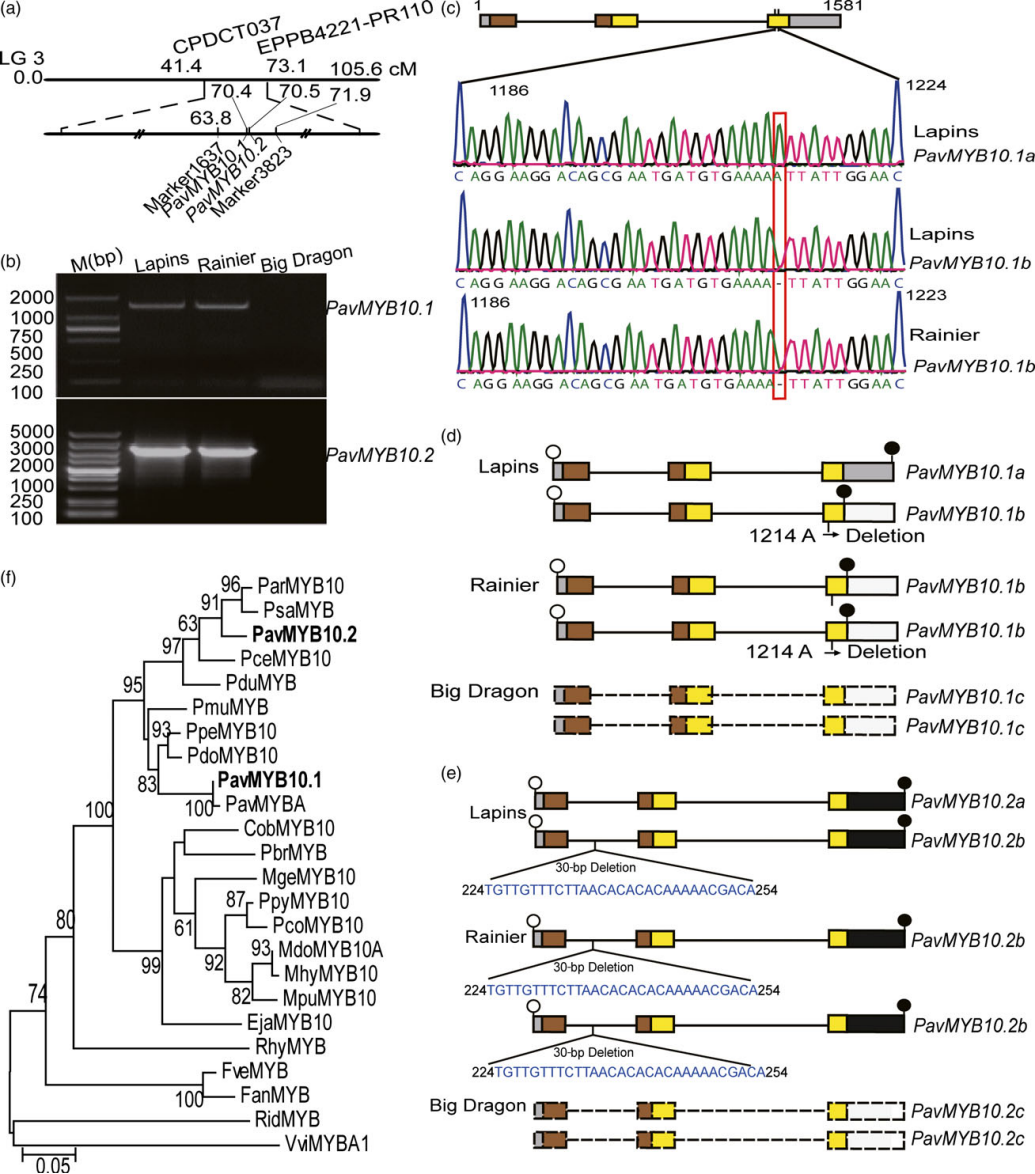
Isolation and analysis of *PavMYB10.1* and *PavMYB10.2* from sweet cherry varieties. (a) Genetic mapping and schematic position of *PavMYB10.1* and *PavMYB10.2* on LG 3. Genetic distance between flanking pairwise molecular markers is shown. *PavMYB10.1* and *PavMYB10.2* were positioned at 70.4 and 70.5 cM between marker 1637 and marker 3823. (b) PCR amplification of the full‐length *PavMYB10.1* and *PavMYB10.2* genes in ‘Big Dragon’, ‘Rainier’ and ‘Lapins’. (c) Sanger sequencing confirmation of heterozygosis and homozygous *PavMYB10.1* genes in ‘Lapins’ and ‘Rainier’. (d and e) The structure of full‐length *PavMYB10.1* and *PavMYB10.2* genes. *PavMYB10.1* and *PavMYB10.2* contain three exons and two introns. A 1‐bp deletion was present in third exon, at position 1214 relative to start codon of *PavMYB10.1b*. Three are 30‐bp deletion in the first intron of *PavMYB10.2b*. Open and filled circles mark start and stop codons. Box represents exons and straight lines represent introns. Sienna and yellow boxes represent R2 and R3 domains. *PavMYB10.1* and *PavMYB10.2* were undetectable in yellow variety ‘Big Dragon’ (named *PavMYB10.1c* and *PavMYB10.2c*). Dashed boxes indicate the *PavMYB10.1c* and *PavMYB10.2c*. (f) Unrooted neighbour‐joining phylogenetic tree of PavMYB10.1, PavMYB10.2 and other MYB transcription factors. Bootstrap values from 1000 replicates are shown at each node.

To identify the molecular mechanism underlying fruit skin colour in sweet cherry, we constructed an F1 population. Because *Prunus avium* exhibits gametophytic self‐incompatibility, the dark‐red variety ‘Lapins’ and the dark‐red variety ‘Wanhongzhu’ were selected as cross‐parents (Sharma *et al*., 2014). Analyses of the F1 hybrids showed that the 415 dark‐red and 150 blush fruit skin phenotypes segregated in a 3 : 1 ratio. This segregation ratio suggested that a single gene with a complete dominance effect explained this trait. An initial locus (70.4–70.5 cM) with the marker 1637 (63.8 cM) and marker 3823 (71.9 cM) was mapped onto sweet cherry LG 3 through a specific‐locus amplified fragment sequencing (SLAF‐seq) analysis (Figure 2a). There were a total of 163 putative genes according to collinearity analysing the peach genome in the region. Interestingly, this region contained *Prupe.3G163100* and *Prupe.3G163300* genes. Blast analyses of the sequence of the genes revealed that they were TFs belonging to the R2R3 MYB family. By homology to the related peach genes, the R2R3 MYB TF genes were named *PavMYB10.1* and *PavMYB10.2*. We identified *PavMYB10.1* and *PavMYB10.2* in three differently coloured sweet cherry varieties.

The *PavMYB10.1* and *PavMYB10.2* genes were detectable in the dark‐red variety ‘Lapins’ and the blush variety ‘Rainier’, but undetectable in the yellow variety ‘Big Dragon’ (Figure 2b). In the Southern blot analyses, positive signals were detected in the *Eco*RI‐digested DNA of ‘Lapins’ and ‘Rainier’, but not in that of ‘Big Dragon’ (Figure S2). The results suggested that a large INDEL is in yellow variety. *PavMYB10.1* was heterozygous in the dark‐red variety ‘Lapins’. They were 1581 and 1580 bp in length. The former and the latter were designated as alleles *PavMYB10.1a* and *PavMYB10.1b*. The *PavMYB10.1* allele was homozygous *PavMYB10.1b* in the blush variety ‘Rainier’. The *PavMYB10.1* gene was undetectable in the yellow variety ‘Big Dragon’ (this allele was called *PavMYB10.1c*). Both *PavMYB10.1a* and *PavMYB10.1b* had three exons and two introns. The two introns were 310 and 599 bp long, and the three exons were 121, 130 and 420 bp (*PavMYB10.1b*) or 421 bp long (*PavMYB10.1a*; Figure S3). In the third exon of *PavMYB10.1b*, a 1‐bp deletion of an adenine was detected at position 1214 relative to the start codon (Figure 2c). The results showed that the dark‐red variety ‘Lapins’ was heterozygous (*PavMYB10.1a* and *PavMYB10.1b*), the blush variety ‘Rainier’ was homozygous for *PavMYB10.1b,* and the yellow variety was homozygous for *PavMYB10.1c* (Figure 2d).

The *PavMYB10.2* was heterozygous in the dark‐red variety in ‘Lapins’. They were 2223 and 2193 bp in length; the former and the latter were designated as alleles *PavMYB10.2a* and *PavMYB10.2b* (Figure S4). The *PavMYB10.2* allele was homozygous *PavMYB10.2b* in the blush variety ‘Rainier’. The *PavMYB10.2 g*ene was undetectable in the yellow variety ‘Big Dragon’ (this allele was called *PavMYB10.2c*). Both *PavMYB10.2a* and *PavMYB10.2b* had three exons and two introns. The three exons were 121, 130 and 484 bp. The two introns were 366 and 1134 bp long (*PavMYB10.2a*), and/or 1104 bp long (*PavMYB10.2b*). The results showed that the dark‐red variety ‘Lapins’ was heterozygous (*PavMYB10.2a* and *PavMYB10.2b*), the blush variety ‘Rainier’ was homozygous for *PavMYB10.2b*, and the yellow variety was homozygous for *PavMYB10.2c* (Figure 2e).

A phylogenetic tree for plant R2R3‐type MYB TFs including PavMYB10.1 and PavMYB10.2 from sweet cherry was constructed by the neighbour‐joining method using full‐length deduced amino acid sequences. The PavMYB10.1 from ‘Lapins’ shared 100% homology with PavMYBA from ‘Hongdeng’, and PavMYB10.2 from ‘Lapins’ 100% homology with PavMYB10 (Figure 2f). Comparison of the deduced amino acid sequence of PavMYB10 with those of several other anthocyanin‐related MYB TFs in other plants revealed a high degree of sequence similarity in the R2 and R3 DNA‐binding domains of these proteins (Figure S5). There were 10 amino acid residue differences in R2R3 domain between PavMYB10.1 and PavMYB10.2. Key amino acid residues of R2R3 domain may play an important role in the transcriptional control of biosynthesis (Heppel *et al*., 2013; Kelemen *et al*., 2015; Zimmermann *et al*., 2004). In the R2 domain, PavMYB10.1 contained an Arg at key position 40, while this residue was Lys in the PavMYB10.2. In the R3 domain, PavMYB10.1 contained a Gly at position 83, while this residue was a Gln in the PavMYB10.2. These results suggested that PavMYB10.1 and PavMYB10.2 may play different roles in anthocyanin biosynthesis.

### Transcript levels of the R2R3‐MYB TFs differ among the three sweet cherry varieties

**Figure 3:**
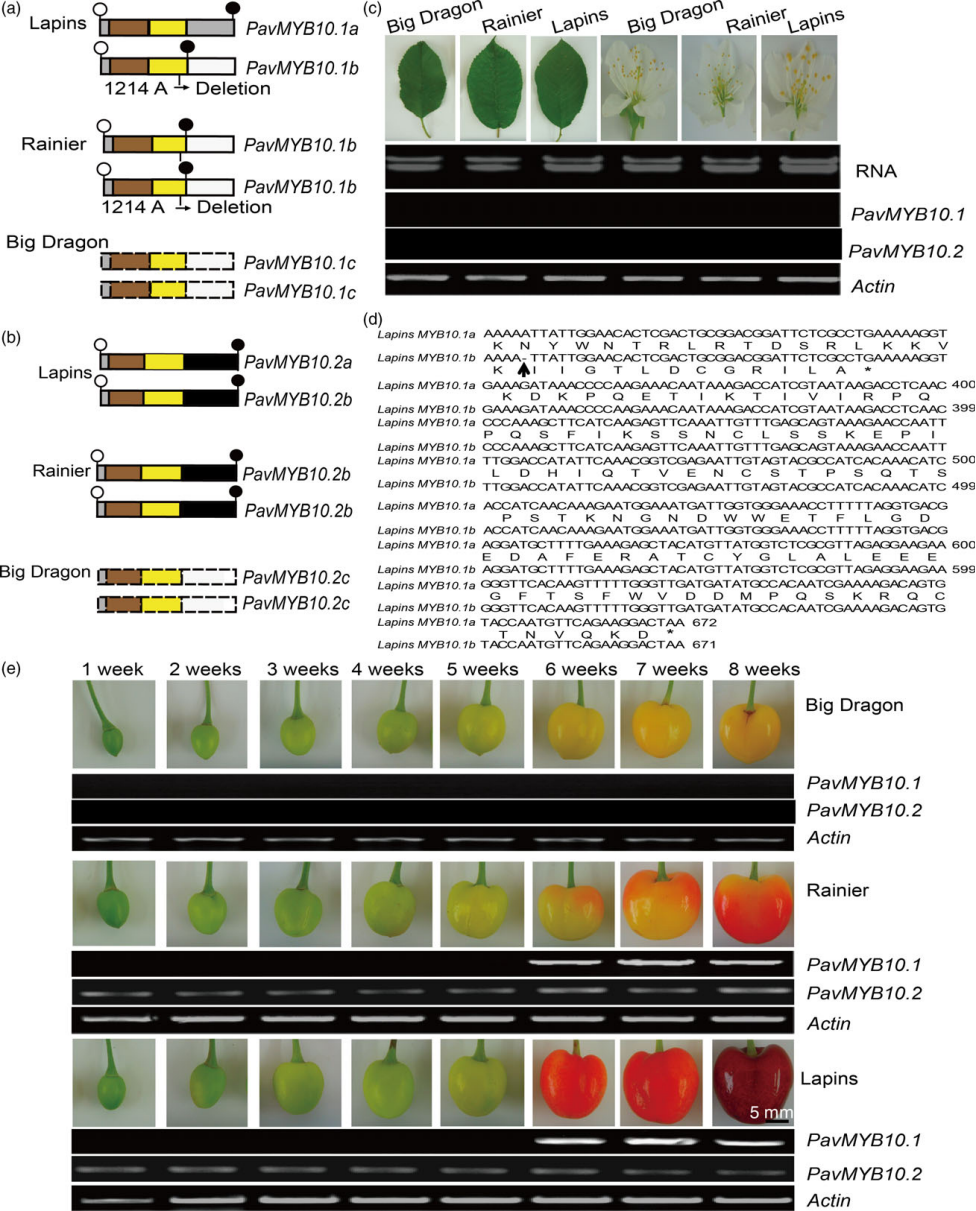
Transcript analysis of *PavMYB10.1* and *PavMYB10.2* in sweet cherry varieties. (a and b) Schematic of *PavMYB10.1* and *PavMYB10.2* transcript structure in ‘Lapins’, ‘Rainier’ and ‘Big Dragon’. Truncated *PavMYB10.1b* transcript was detectable in blush variety ‘Rainier’. *PavMYB10.1a* and *PavMYB10.1b* transcripts were detected in dark‐red variety ‘Lapins’. *PavMYB10.2a* and *PavMYB10.2b* were identical in ‘Lapins’ and ‘Rainier’. Dashed boxes indicate undetectable *PavMYB10.1* and *PavMYB10.2*. Open and filled circles represent start and stop codons. Sienna and yellow boxes represent R2 and R3 domains. (c) Difference of the *PavMYB10.1a* and *PavMYB10.1b* sequence. Upwards arrow indicates position of deletion, asterisk marks stop codon. (d) RT‐PCR analysis of *PavMYB10.1* and *PavMYB10.2* transcript levels in leaves and flowers of ‘Big Dragon’, ‘Rainier’ and ‘Lapins’. *Actin* was used as loading control. (e) RT‐PCR analysis of *PavMYB10.1* and *PavMYB10.2* transcript levels during fruit development of ‘Big Dragon’, ‘Rainier’ and ‘Lapins’. Transcript levels of *PavMYB10.1* and *PavMYB10.2* from 1 to 8 weeks after full bloom in ‘Big Dragon’, ‘Rainier’ and ‘Lapins’ are shown. *Actin* was used as a loading control.

Because the *PavMYB10.1* and *PavMYB10.2* genes of ‘Big Dragon’, ‘Rainier’ and ‘Lapins’ differed at the genomic DNA level, they were transcribed at different levels among the three varieties (Figure 3a,b). *PavMYB10.1* and *PavMYB10.2* transcripts were undetectable in the leaves and flowers of the three varieties (Figure 3c). The ripe fruit of ‘Lapins’ contained the 672‐bp full‐length *PavMYB10.1a* and the 671‐bp full‐length *PavMYB10.1b* cDNAs with open reading frames encoding 223 and 113 amino acids, respectively. The 671‐bp full‐length *PavMYB10.1b* cDNA from the blush variety ‘Rainier’ contained an open reading frame encoding 113 amino acids and a stop codon at position 342 (Figure 3d). The cDNA sequence analysis revealed that there was a 1‐bp adenine deletion in *PavMYB10.1b* in the blush variety ‘Rainier’ at position 305 (Figure S6). Fruit of the dark‐red variety ‘Lapins’ contained both intact *PavMYB10.1a* and *PavMYB10.1b* transcripts. *PavMYB10.2a* and *PavMYB10.2b* were same 738‐bp full‐length cDNAs with open reading frames encoding 245 amino acids (Figure S7). The transcript analyses showed that *PavMYB10.1* and *PavMYB10.2* transcripts were absent from the yellow variety ‘Big Dragon’.

To clarify the transcription pattern of *PavMYB10.1* and *PavMYB10.2* during fruit development from 1 to 8 weeks after full bloom, RT‐PCR analyses were performed to analyse transcript levels in the three varieties. The transcript levels of *PavMYB10.1* in fruits of ‘Lapins’ and ‘Rainier’ increased rapidly from 6 to 8 weeks. *PavMYB10.1* was specifically expressed in fruit at 6, 7 and 8 weeks in the dark‐red variety ‘Lapins’ and the blush variety ‘Rainier’. The transcript levels of *PavMYB10.2* in fruits of ‘Lapins’ and ‘Rainier’ were highly expressed from 1 to 8 weeks. No *PavMYB10.1* and *PavMYB10.2* transcripts were detected in fruit development of the yellow variety ‘Big Dragon’ (Figure 3e). These results indicated that the specific expression of *PavMYB10.1* and *PavMYB10.2* depended on the tissue and the stage of ripeness. The expression analysis of *PavMYB10.1* and *PavMYB10.2* genes showed that the transcript levels of the regulatory gene *PavMYB10.1* were nearly consistent with those of anthocyanin content and that the transcript levels of the *PavMYB10.2* were high during the fruit development in dark‐red and blush sweet cherry. In addition, the *PaMYB10.2* (*PavMYB10.2a* and *PavMYB10.2b*) from dark‐red and blush varieties has same cDNA sequences. The results suggested that *PavMYB10.1* correlates with fruit coloration.

### *PavMYB10.1* expression is positively correlated with anthocyanin accumulation and with transcript levels of *PavANS* and *PavUFGT*

**Figure 4:**
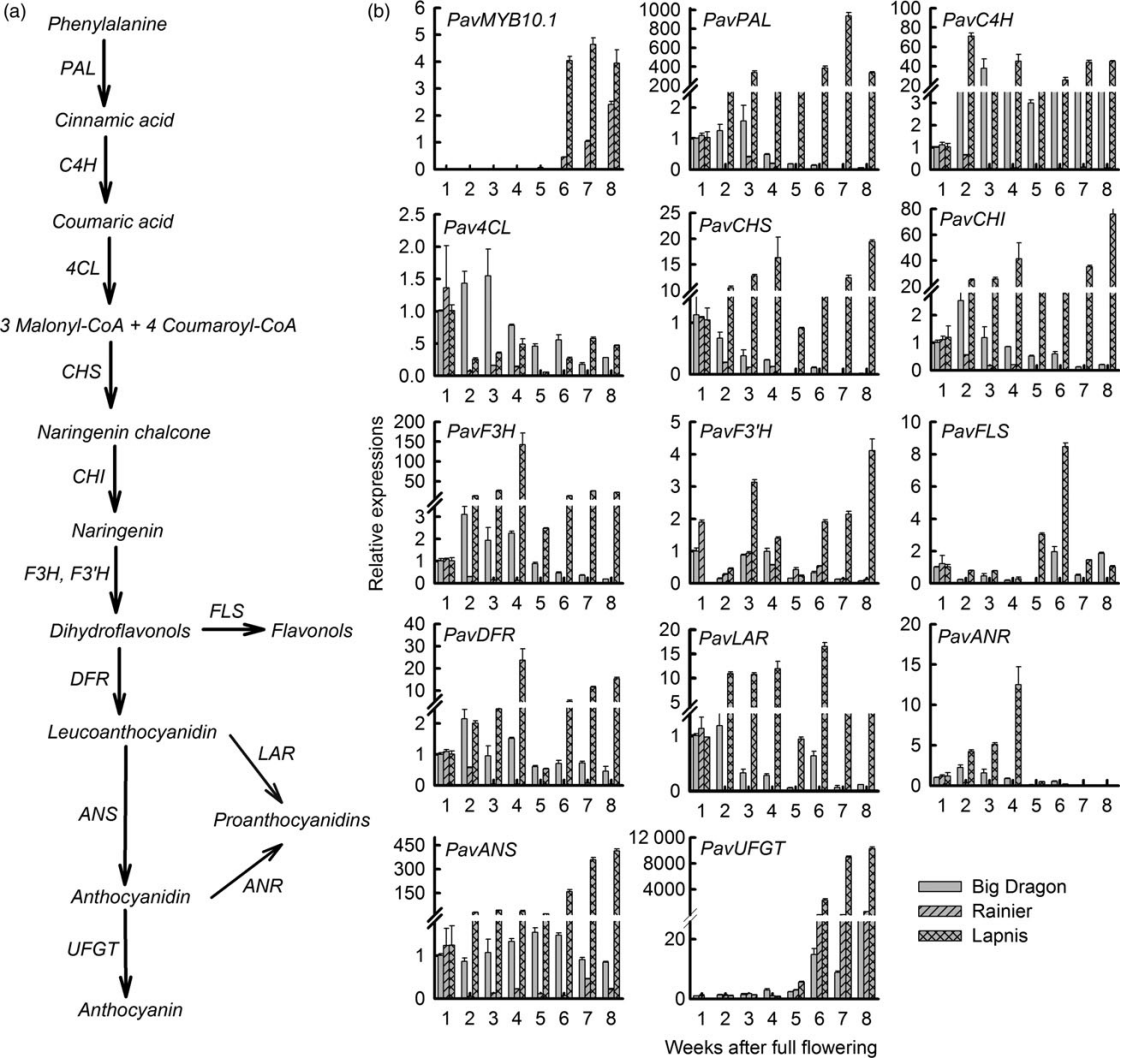
A model of anthocyanin synthesis pathway and transcription factor *PavMYB10.1* and structural gene expressions during sweet cherry fruit development. (a) A model of anthocyanin synthesis pathway. Structural genes for each step are indicated as follows: *PAL*, phenylalanine ammonia lyase; *C4H*, cinnamate 4‐hydroxylase; *4CL*, 4‐coumarate‐CoA ligase; *CHS*, chalcone synthase; *CHI*, chalcone isomerase; *F3H*, flavanone 3‐hydroxylase; *F3′H*, flavonoid 3′‐hydroxylase; *DFR*, dihydroflavonol 4‐reductase; *ANS*, anthocyanidin synthase; *UFGT*, flavonoid‐3‐O‐glucosyltransferase; *FLS*, flavonol synthase; *LAR*, leucoanthocyanidin reductase; *ANR*, anthocyanidin reductase. (b) Relative expressions of *PavMYB10.1* and structural genes during sweet cherry fruit development.

Anthocyanins, flavonols and proanthocyanidins are synthesized via the flavonoid biosynthetic pathway (Figure 4a). Anthocyanins are synthesized by several structural genes. Among these genes, *PAL*,* C4H*,* 4CL*,* CHS*,* CHI*,* F3H* and *F3′H* are common upstream genes of the anthocyanin, flavonol and proanthocyanin branch pathways. Striking differences in gene transcription were observed during fruit development in these three varieties (Figure 4b). In the dark‐red variety ‘Lapins’, the transcript levels of the upstream genes common to anthocyanin, flavonol and proanthocyanin branch pathways (*PavPAL*,* PavC4H*,* Pav4CL*,* PavCHS*,* PavCHI*,* PavF3H*) were very low at the beginning of fruit development, strongly increased up to 3 or 4 weeks, then decreased again and strongly increased when the fruit colour changed to deep red from 6 to 8 weeks. In contrast, the transcript levels of *PavPAL*,* PavCHS*,* PavCHI* and *PavF3H* were very low during fruit development in the yellow cultivar ‘Big Dragon’ and the blush cultivar ‘Rainier’. Among these enzymes, ANS and UFGT are the last two steps in the anthocyanin synthesis pathway. We analysed the transcript levels of *PavANS* and *PavUFGT* and observed striking differences among the three sweet cherry varieties during fruit development. In the dark‐red variety ‘Lapins’, the transcript levels of *PavANS* and *PavUFGT* were very low at the beginning of fruit development and strongly increased when the fruit colour changed to dark red from 6 to 8 weeks. In contrast, the transcript levels of *PavANS* and *PavUFGT* remained very low throughout fruit development in the yellow variety ‘Big Dragon’. The transcript levels of the regulatory gene *PavMYB10.1* were nearly consistent with those of *PavANS* and *PavUFGT*. Proanthocyanidins are synthesized from cyanidin by anthocyanidin reductase (ANR). There were high transcript levels of *PavANR* in the green fruit. The results showed that there were two stages during fruit development; the proanthocyanin pathway was the main pathway in green fruit, and the anthocyanin pathway was the main pathway in colouring fruit.

The correlations between anthocyanin content and the relative transcript levels of *PavMYB10.1* and the anthocyanin structural genes (*PavANS* and *PavUFGT*) differed among the three varieties (Tables S1 and S2). In the dark‐red variety ‘Lapins’, there were significant positive correlations between anthocyanin content and *PavMYB10.1* transcript levels (*P *= 0.014 < 0.05), between *PavMYB10.1* and *PavANS* transcript levels (*P *= 0.002 < 0.05), and between *PavMYB10.1* and *PavUFGT* transcript levels (*P *= 0.006 < 0.05). In the blush variety ‘Rainier’, there were significant positive correlations between anthocyanin content and transcript levels of *PavMYB10.1* (*P *= 0.002 < 0.05), and between *PavMYB10.1* and *PavUFGT* transcript levels (*P *= 0.000 < 0.05). In the yellow variety ‘Big dragon’, which lacked *PavMYB10.1* expression, no such correlations were found. *PavANS* and *PavUFGT* encode the key enzymes catalysing the last two steps in the anthocyanin synthesis pathway. The results showed that the transcript level of *PavMYB10.1* was positively correlated with anthocyanin accumulation and with *PavANS* and *PavUFGT* transcript levels in cherry fruits.

### Role of *PavMYB10.1* in anthocyanin biosynthesis pathway

**Figure 5:**
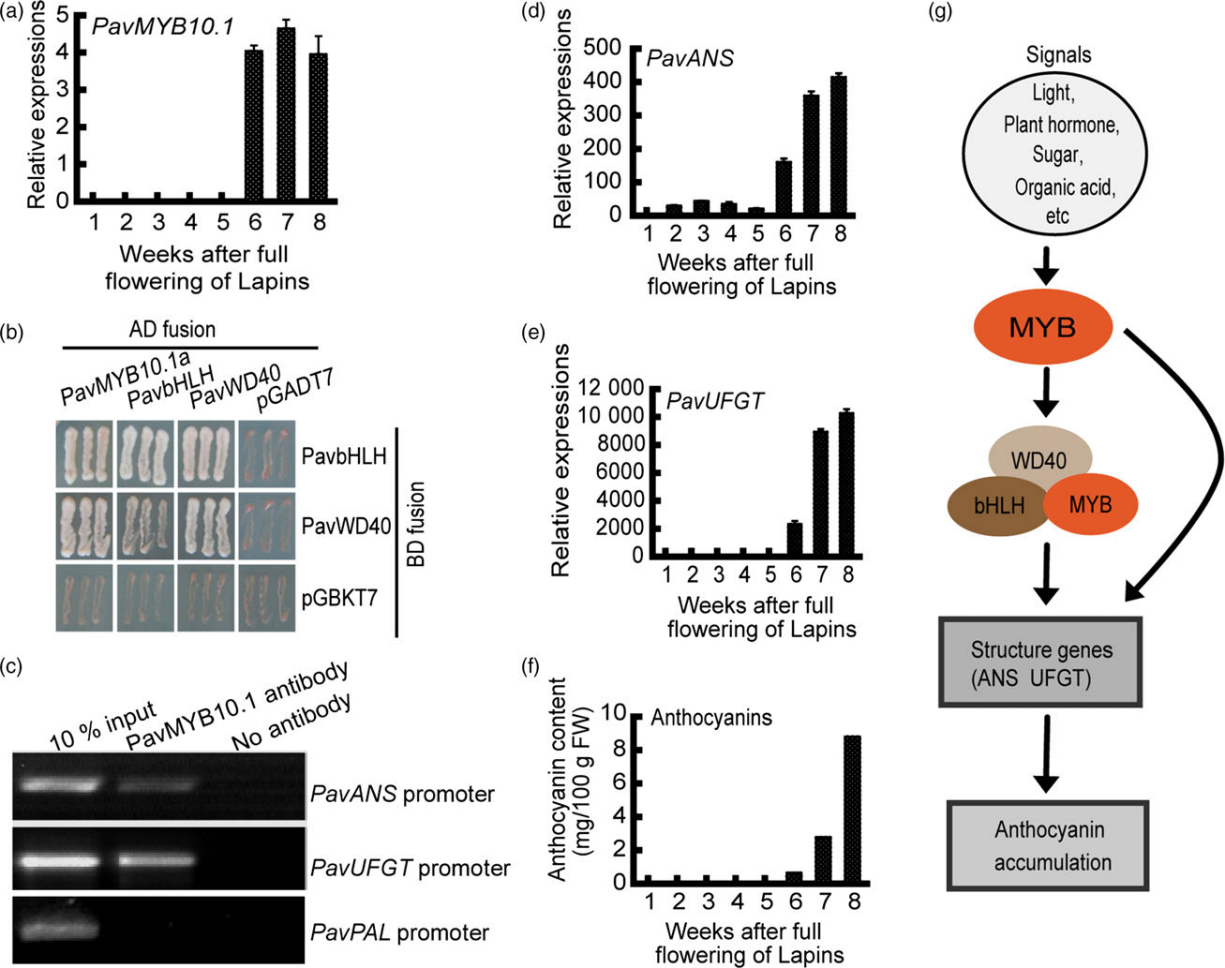
Role of PavMYB10.1 regulators in transcriptional activation of structural genes and anthocyanin accumulation. (a) Relative transcript levels of *PavMYB10.1* in dark‐red variety ‘Lapins’ during fruit development, as determined by qRT‐PCR. (b) Interaction of PavMYB10.1a with PavbHLH and PavWD40. (c) PavMYB10.1 was selectively recruited to *PavANS*, *PavUFGT* and *PavPAL* promoter regions in dark‐red variety ‘Lapins’, as determined by ChIP assay. (d) and (e) Relative transcript levels of *PavANS* and *PavUFGT* in developing fruit of dark‐red variety ‘Lapins’, as determined by qRT‐PCR. (f) Changes in anthocyanin contents during fruit development in dark‐red variety ‘Lapins’. (g) A model depicting role of MYB regulators in transcriptional activation of structural genes and anthocyanin accumulation.

MYB TFs activate specific parts of the anthocyanin pathway and respond differentially to signals such as plant hormones, sugars and light cues (Giliberto *et al.,* 2005, Heijde *et al.,* 2013, Shen *et al*., 2014). The transcription of structural genes is regulated by MYB–bHLH–WD40 (MBW) complexes or MYB TFs. High transcript levels of the structural genes *PavANS* and *PavUFGT* were related to increased anthocyanin accumulation (Figure 5). In the dark‐red variety ‘Lapins’, the transcript levels of *PavMYB10.1* increased rapidly from 6 to 8 weeks (Figure 5a). *PavMYB10.1*, as a TF, is a cytoplasm‐ and nucleus‐localized protein (Figure S8). We performed yeast two‐hybrid assays and observed that PavMYB10.1a interacted with PavbHLH and PavWD40 (Figure 5b), but PavMYB10.1b did not interact with PavbHLH or PavWD40 (Figure S9). Chromatin immunoprecipitation (ChIP) assays showed that PavMYB10.1a was selectively recruited to the *PavANS* and *PavUFGT* promoter regions containing AE‐box light‐responsive elements (AGAAACAA; Figure 5c). The transcript levels of *PavANS* and *PavUFGT* increased rapidly from 6 to 8 weeks (Figure 5d,e), and anthocyanin contents also increased rapidly from 6 to 8 weeks (Figure 5f). MYB10.1 formed a putative canonical MBW activation complex with bHLH and WD40. The MBW complex was selectively recruited to the *ANS* and *UFGT* promoter regions and activated their transcription. Anthocyanin accumulation was related to high transcript levels of *ANS* and *UFGT* (Figure 5g). Together, these analyses revealed the mechanism by which *PavMYB10.1* controls anthocyanin biosynthesis and fruit coloration in sweet cherry.

### Inheritance of *PavMYB10.1* alleles is consistent with fruit skin colour traits

To study the inheritance of fruit skin colour traits, three crosses were performed in 2007, and the fruit colours of the progeny were investigated in 2012. The cross between the dark‐red variety ‘Lapins’ (*PavMYB10.1a*/*PavMYB10.1b*) and the dark‐red variety ‘Wanhongzhu’ (*PavMYB10.1a*/*PavMYB10.1b*) produced an F1 population of 565 individuals, with 415 dark‐red (*PavMYB10.1a*/*PavMYB10.1a* and *PavMYB10.1a*/*PavMYB10.1b*) and 150 blush (*PavMYB10.1b*/*PavMYB10.1b*) individuals. Analyses of the F1 hybrids showed that the dark‐red and the blush fruit phenotypes segregated in a 3 : 1 ratio (χ^2^ = 0.723), compatible with Mendel's first segregation law. The cross between the dark‐red variety ‘Lapins’ (*PavMYB10.1a*/*PavMYB10.1b*) and the blush variety ‘Hongyan’ (*PavMYB10.1b*/*PavMYB10.1b*) produced an F1 population of 57 individuals, with 32 dark‐red (*PavMYB10.1a*/*PavMYB10.1b*) individuals and 25 blush (*PavMYB10.1b*/*Pav MYB10.1b*) individuals (a segregation ratio of 1 : 1; χ^2^ = 0.860). The cross between the blush variety ‘Rainier’ (*PavMYB10.1b*/*PavMYB10.1b*) and the blush variety ‘Hongmi’ (*PavMYB10.1b*/*PavMYB10.1b*) produced 162 (*PavMYB10.1b*/*PavMYB10.1b*) F1 individuals, all with the blush fruit phenotype (Table 1). When the *PavMYB10.1* genes were evaluated in all individuals, the dark‐red individuals had at least one intact *PavMYB10.1a* and the blush ones had only *PavMYB10.1b*.

**Table 1:** Inheritance of fruit skin colour trait in sweet cherry crosses

Female	Male	Year of crossing	Year of bearing fruit	No. of dark‐red F1 progeny	No. of blush F1 progeny	Ratio	χ^2^
Lapins	Wanhongzhu	2007	2012	415	150	3 : 1	0.723
Lapins	Hongyan	2007	2012	32	25	1 : 1	0.860
Rainier	Hongmi	2007	2012	0	162	0 : 1	0

### *PavMYB10.1* determines fruit skin colour

**Figure 6:**
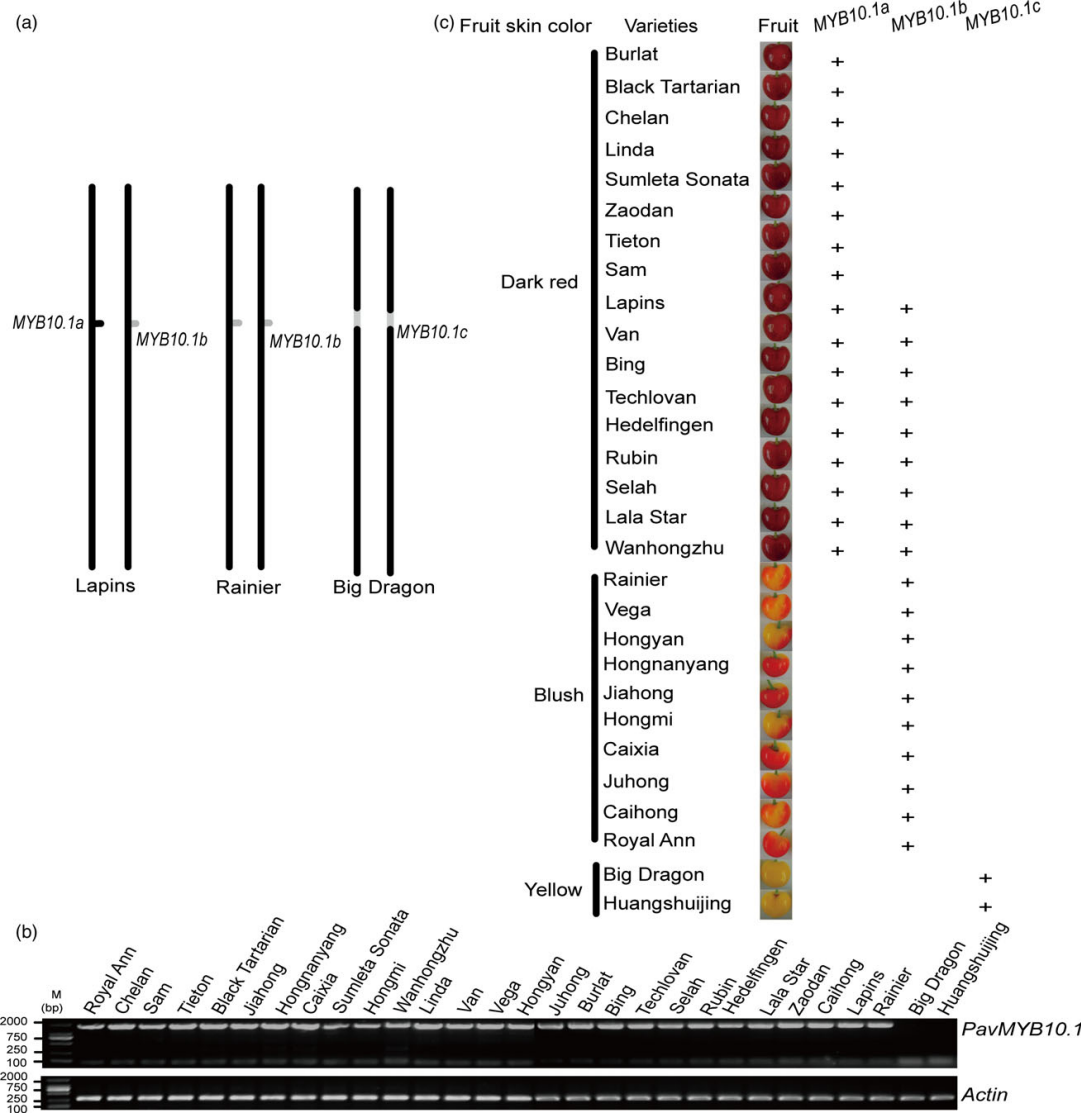
Correlation between *PavMYB10.1* and fruit skin colour. (a) Different alleles of *PavMYB10.1* on chromosome in sweet cherry varieties ‘Big Dragon’, ‘Rainier’ and ‘Lapins’. Black dot indicates *PavMYB10.1a*; grey dot indicates *PavMYB10.1b*; and incised grey site indicates *PavMYB10.1c*. (b) PCR‐amplified *PavMYB10.1* from genomic DNA of different sweet cherry varieties. *Actin* was used as a loading control for DNA samples from different sweet cherry varieties. (c) Correlation between the presence of *PavMYB10.1* and fruit colour. Dark‐red varieties ‘Burlat’, ‘Black Tartarian’, ‘Chelan’, ‘Linda’, ‘Sumleta Sonata’, ‘Zaodan’, ‘Tieton’ and ‘Sam’ contained only *PavMYB10.1a*. Dark‐red varieties ‘Lapins’, ‘Van’, ‘Bing’, ‘Techlovan’, ‘Hedelfingen’, ‘Rubin’, ‘Selah’, ‘Lala Star’ and ‘Wanhongzhu’ contained *PavMYB10.1a* and *PavMYB10.1b*. Blush varieties ‘Rainier’, ‘Vega’, ‘Hongnanyang’, ‘Jiahong’, ‘Hongmi’, ‘Caixia’, ‘Juhong’, ‘Caihong’, ‘Royal Ann’ and ‘Hongyan’ contained only *PavMYB10.1b*. *PavMYB10.1c* was in yellow varieties ‘Big Dragon’ and ‘Huangshuijing’. ‘+’ indicates *PavMYB10.1a*, *PavMYB10.1b* and *PavMYB10.1c*.

The *PavMYB10.1* gene differed among the three sweet cherry varieties (Figure 6a). We analysed *PavMYB10.1* in 29 varieties (2 yellow varieties, 9 blush varieties and 18 dark‐red varieties; Table 2), to determine whether this gene could be used to distinguish yellow, blush and dark‐red sweet cherry varieties. The *PavMYB10.1* genes were amplified by PCR from the blush and dark‐red sweet cherry varieties, but not from the two yellow varieties (Figure 6b). Sequence analyses revealed that the blush sweet cherry varieties ‘Rainier’, ‘Vega’, ‘Hongnanyang’, ‘Jiahong’, ‘Hongmi’, ‘Caixia’, ‘Juhong’, ‘Caihong’, ‘Hongyan’ and ‘Royal Ann’ had homozygous *PavMYB10.1b* genes, while the dark‐red sweet cherry varieties ‘Burlat’, ‘Black Tartarian’, ‘Chelan’, ‘Linda’, ‘Sumleta Sonata’, ‘Zaodan’, ‘Tieton’ and ‘Sam’ had two intact *PavMYB10.1a* genes. The dark‐red sweet cherry varieties ‘Lapins’, ‘Van’, ‘Bing’, ‘Techlovan’, ‘Hedelfingen’, ‘Rubin’, ‘Selah’, ‘Lala Star’ and ‘Wanhongzhu’ had the intact *PavMYB10.1a* and *PavMYB10.1b*. According to differences in their *PavMYB10.1* alleles, the sweet cherry varieties were divided into three types: *PavMYB10.1c* gene (yellow varieties such as ‘Huangshuijing’ and ‘Big Dragon’), homozygous for *PavMYB10.1b* (blush varieties such as ‘Royal Ann’, ‘Hongnanyang’, and ‘Jiahong’) and intact *PavMYB10.1a* (dark‐red varieties; Figure 6c). These results confirmed that the *PavMYB10.1* gene is a reliable DNA marker to select differently coloured sweet cherry varieties.

**Table 2:** Origin, reference and fruit skin colour of sweet cherry varieties used in this study

Accession	Parentage	Origin and reference	Fruit skin colour
Big Dragon	Unknown	China	Yellow
Bing	Black Republican op	USA (Brettin *et al*., 2000)	Dark red
Black Tartarian	Unknown	Unknown (Fernandez i Martí *et al*., 2012)	Dark red
Burlat	Unknown	France (Lisek and Rozpara, 2009)	Dark red
Caihong	Unknown	China (Zhang *et al*., 2012a)	Blush
Caixia	Unknown	China (Zhang *et al*., 2012b)	Blush
Chelan	Stella × Beaulieu	USA (Godini *et al*., 2009)	Dark red
Jiahong	Bing × Vega	China	Blush
Juhong	Napoleon × Governor Wood	China	Blush
Hedelfingen	Unknown	Germany (Lisek and Rozpara, 2009)	Dark red
Hongnanyang	Unknown	China	Blush
Hongmi	Napoleon × Governor Wood	China	Blush
Hongyan	Napoleon × Governor Wood	China	Blush
Huangshuijing	Unknown	China	Yellow
Lala Star	Compact Lambert × Lapins	Italy (Fernandez i Martí *et al*., 2012)	Dark red
Lapins	Van × Stella	Canada (Fernandez i Martí *et al*., 2012)	Dark red
Linda	Stella × Early Burlat	Hungary (Brózik, 1993)	Dark red
Rainier	Bing × Van	USA (Granger *et al*., 1993)	Blush
Royal Anne	Unknown	Germany (Cabrera *et al*., 2012)	Blush
Robin	Unknown	Canada (Fernandez i Martí *et al*., 2012)	Dark red
Sam	Windsor × unknown	Canada(Fernandez i Martí *et al*., 2012)	Dark red
Selah	(Rainier × Bing) × Stella	USA (Cabrera *et al*., 2012)	Dark red
Sumleta Sonata	Lapins × (Van × Stella)	Canada (Kappel *et al*., 2000)	Dark red
Techlovan	Van × Kordia	Czech Republic (Lisek and Rozpara, 2009)	Dark red
Tieton	Stella × Early Burlat	USA (Cabrera *et al*., 2012)	Dark red
Van	Empress Eugenie op	Canada (Brettin *et al*., 2000)	Dark red
Vega	Bing × Victor	Canada (Fernandez i Martí *et al*., 2012)	Blush
Wanhongzhu	Unknown	China	Dark red
Zaodan	‘Xesphye’ mutant	China (Yan *et al*., 2012)	Dark red

## Discussion

### Inducible *MYB10.1* and its signals

Many reports show that the expression of TFs MYB is inducible by plant hormone‐related and environmental factors (especially light) in anthocyanin biosynthesis during fruit ripening (Shen *et al*., 2014; Vimolmangkang *et al*., 2014). Studies on the signalling mechanism behind light‐related anthocyanin biosynthesis in fruits have markedly increased recently. Light can regulate expression of genes in the anthocyanin biosynthesis pathway by inducing TFs MYB (Takos *et al*., 2006; Vimolmangkang *et al*., 2014). Abscisic acid (ABA) is an important hormone associated with fruit maturation processes, such as sugar accumulation and softening, in sweet cherry (Ren *et al*., 2010). ABA treatment significantly induced anthocyanin accumulation, while treatment with the ABA biosynthesis inhibitor nordihydroguaiaretic acid (NDGA) blocked anthocyanin production. *PavMYBA* expression was up‐regulated by ABA, but down‐regulated by NDGA treatment. ABA is a signal molecule that promotes accumulating anthocyanin in the red‐coloured sweet cherry (Shen *et al*., 2014). Our results show that expressions of *PavMYB10.1* increase rapidly at fruit coloration stage of ‘Lapins’ and ‘Rainier’, while it is very low in the leaves, flowers and green fruit stage of the three varieties (Figure 3). This indicates the specific expression of *PavMYB10.1* depended on the tissue and the stage of ripeness. It can be presumed that light and ABA signals may have important roles in inducing *PavMYB10.1*.

ANS and UFGT catalyse the last two steps in the anthocyanin synthesis pathway. Proanthocyanidins are synthesized from cyanidin by ANR. In strawberry, transcript levels were measured for key structural genes involved in both proanthocyanin and anthocyanin biosyntheses. The results showed that the flavonoids are mainly represented by proanthocyanidins, while in ripe fruits the red‐coloured anthocyanins also accumulate (Schaart *et al*., 2013). A similar case is also observed in this study. The transcript levels of *PavANS* and *PavUFGT* were very low at the beginning of fruit development and strongly increased when the fruit colour changed to dark red from 6W to 8W. There were high transcript levels of *PavANR* in the green fruits (Figure 4). Our results suggest that there are two stages during fruit development; the proanthocyanin accumulation is the main pathway in green fruits, and the anthocyanin accumulation is the main pathway in ripe fruits.

### MYB, bHLH and WD40 interaction

Anthocyanin pathway genes are known to be coordinately induced and TFs that regulate the expression of the structural genes of the pathway have been identified in several species. Regulation of the pathway occurs by the MBW complex from interaction of R2R3 MYB TF, bHLH and WD40‐repeat proteins (D'Amelia *et al*., 2014; Espley *et al*., 2007; Gonzalez *et al*., 2008; Schaart *et al*., 2013). In apple, the interaction between MdMYB and MdbHLH proteins activates transcription of DFR by transient assays (Espley *et al*., 2007). Here, we show that PavMYB10.1a interacts with PavbHLH and PavWD40, but PavMYB10.1b does not interact with PavbHLH or PavWD40 in sweet cherry (Figure S9). PavMYB10.1a forms a putative canonical MBW activation complex with PavbHLH and PavWD40. The MBW complex is selectively recruited to the *PavANS* and *PavUFGT* promoter regions containing AE‐box light‐responsive elements (AGAAACAA) of *PavANS* and *PavUFGT* (Roy *et al*., 2012). Anthocyanin contents also increase rapidly in fruit coloration. PavMYB10.1b does not form a putative canonical MBW and slowly increases anthocyanin accumulation. In this scenario, a 1‐bp deletion of an adenine could cause an alteration of the interaction between MYB proteins with the copartners, resulting in a variety of anthocyanin accumulation. Our results confirm the previous findings that it is the MYB component from the MYB–bHLH–WD40 protein complex primarily responsible for anthocyanin accumulation.

### R2R3 MYB TFs determine fruit colours

In many different fruits, the R2R3 MYB TFs control the expression of structural genes in the anthocyanin biosynthesis pathway. *MYB* genes and their promoters play an important role in anthocyanin accumulation in fruit. Three research groups have independently identified an R2R3 MYB TF responsible for anthocyanin accumulation in apple (*M. domestica*) fruit—the loci were named *MdMYB1*,* MdMYB10* and *MdMYBA* (Allan *et al*., 2008; Ban *et al*., 2007; Espley *et al*., 2007; Lin‐Wang *et al*., 2010; Takos *et al*., 2006). The coding region of *MdMYBA* showed 100% and 98% similarity to those of *MdMYB1* and *MdMYB10* (Ban *et al*., 2007). In addition, *MdMYB10* and *MdMYBA* were mapped to the same region on LG 9 (Ban *et al*., 2007; Chagné *et al*., 2007). Subsequent experiments have shown that *MdMYB1*,* MdMYB10* and *MdMYBA* are likely to be allelic (Lin‐Wang *et al*., 2010; Telias *et al*., 2011). In apple, an allelic rearrangement in the promoter of *MdMYB10* was shown to regulate *MdMYB10* transcript levels and the subsequent ectopic accumulation of anthocyanins (Espley *et al*., 2007). In grape (*V*. *vinifera*), the colour of berry skins is determined by anthocyanin accumulation (Fournier‐Level *et al*., 2010). White‐skinned grape varieties are thought to have arisen from different red varieties by independent mutations (Walker *et al*., 2007). *Myb*‐related genes regulate anthocyanin biosynthesis in grape. Kobayashi found that genomic *VvmybA1* was homozygous in ‘Italia’, but heterozygous in ‘Ruby Okuyama’. The heterozygous alleles, *VvmybA1a* and *VvmybA1b*, differed in their 5′‐flanking regions but were identical in their coding sequences (Kobayashi *et al*., 2004; Walker *et al*., 2007). *VvmybA1a* contained a retrotransposon, designated as *Gret1* (grapevine retrotransposon), and located upstream of the *VvmybA1*‐coding sequence. Mutations caused by retrotransposon insertions in or near genes can alter gene expression or the structure of the encoded proteins (Kobayashi *et al*., 2004; Walker *et al*., 2007).

In sweet cherry, fruit skin colours vary widely because of differences in pigment profiles. Anthocyanins are responsible for the red colour of sweet cherry fruit skins. Different research groups have independently identified an R2R3 MYB TF responsible for anthocyanin accumulation in sweet cherry fruit. These loci, which are allelic in sweet cherry, have been named *PavMYB1*,* PavMYB10* and *PavMYBA* (Lin‐Wang *et al*., 2010; Shen *et al*., 2014; Sooriyapathirana *et al*., 2010; Starkevič *et al*., 2015). The coding region of *PavMYB10.1* showed 100% similarity to those of *PavMYB1* and *PavMYBA*. In addition, *PavMYB10.2* showed 100% similarity to those of *PavMYB10* (Lin‐Wang *et al*., 2010; Starkevič *et al*., 2015). Starkevič isolated and analysed several closely related *MYB10* genes from different cultivars of sweet cherry. Their results indicated that transcription level of variant PaMYB10.1 correlates with fruit coloration (Starkevič *et al*., 2015). In this study, *PavMYB10.1* and *PavMYB10.2* were mapped to the same region on LG 3 (Figure 1). *PavMYB10.1* regulates the subsequent ectopic accumulation of anthocyanins. *PavMYB10.1* controls sweet cherry fruit skin coloration. *PavMYB10.2* was not correlated with anthocyanin accumulation. *PavMYB10.1* controlled *PavANS* and *PavUFGT* expression and led to anthocyanin accumulation in sweet cherry fruit. Based on their genomic *PavMYB10.1* sequences, sweet cherry varieties could be divided into three groups based on their fruit skin colour: yellow skin (*PavMYB10.1c*), blush (homozygous for *PavMYB10.1b*) and red (at least one intact *PavMYB10.1a*; Figure S1).

Together, the results of this study showed that the skin colour of sweet cherry fruit is controlled by the three *PavMYB10.1* alleles. *PavMYB10.1a* was dominant to *PavMYB10.1b* and *PavMYB10.1b* was dominant to *PavMYB10.1c* in diploid sweet cherry. The allele *PavMYB10.1a* was present in individuals with dark‐red fruit, but not in those with yellow fruit, while the allele *PavMYB10.1b* was present in individuals with blush fruit, but not in those with yellow fruit. All individuals that produced yellow fruit had only the *PavMYB10.1c* allele. Therefore, *PavMYB10.1a*/*PavMYB10.1a*,* PavMYB10.1a*/*PavMYB10.1b* and *PavMYB10.1a*/*PavMYB10.1c* are leading for red fruit; *PavMYB10.1b*/*PavMYB10.1b* and *PavMYB10.1b*/*PavMYB10.1c* are leading for blush fruit; and *PavMYB10.1c*/*PavMYB10.1c* is leading for yellow fruit. These results, which demonstrate the involvement of *PavMYB10.1* in the regulation of anthocyanin biosynthesis, will be useful for the development of biotechnological tools to generate new sweet cherry varieties with enhanced anthocyanin content or improved fruit colours.

In summary, we demonstrate that *PavMYB10.1* plays a key role in regulating anthocyanin biosynthesis in sweet cherry. Different alleles confer the different fruit colours of sweet cherry varieties. The specific expression of *PavMYB10.1* depended on the tissue and the stage of ripeness. PavMYB10.1a forms a putative canonical MBW activation complex with PavbHLH and PavWD40. The MBW complex is selectively recruited to the *PavANS* and *PavUFGT* promoter regions containing AE‐box light‐responsive elements (AGAAACAA) for improving anthocyanin accumulation. Inheritance of colour traits is determined by a single gene *PavMYB10.1*, which had three alleles *PavMYB10.1a*,* PavMYB10.1b* and *PavMYB10.1c*. Different alleles confer the different fruit colours of sweet cherry varieties. These findings not only provide insight into the molecular mechanism of anthocyanin biosynthesis, but also have implications for the development of new varieties through classical breeding or a biotechnological approach.

## Experimental procedures

### Sweet cherry crosses

To study the inheritance of colour traits, three crosses were conducted in 2007: dark‐red variety ‘Lapins’ × dark‐red variety ‘Wanhongzhu’; dark‐red variety ‘Lapins’ × blush variety ‘Hongyan’; and blush variety ‘Rainier’ × blush variety ‘Hongmi’. The fruit colours of the progeny were investigated 5 years later.

### R2R3‐MYB TF genes mapping

An F1 population with 565 plants was generated from a cross between the dark‐red variety ‘Lapins’ and the dark‐red variety ‘Wanhongzhu’. From the total population, a linkage mapping subset of 565 individuals was selected. An initial scan with marker CPDCT037 and EPPB4221‐PR110 mapped this locus onto sweet cherry LG 3. Two new specific‐locus amplified fragment sequencing (SLAF‐seq) markers (marker 1637 and marker 3823) were developed, and the site of the locus was identified based on the results of BLAST peach genome analyses (Dirlewanger *et al*., 2004; Verde *et al*., 2013; Wang *et al*., 2015a,b).

### Sample collection

The sweet cherry varieties ‘Big Dragon’, ‘Rainier’, ‘Lapins’ and others were cultivated under field conditions at the Institute of Forestry and Pomology, Beijing Academy of Agriculture and Forestry Sciences, Beijing, China (Table 2). The samples included leaves, flowers and fruits at different developmental stages (1–8 weeks after full bloom; 1–8 weeks). Mixtures of skin and flesh were used as the samples in all experiments. Samples were frozen in liquid nitrogen and stored at −80 °C until analysis.

### Anthocyanin determination

Total anthocyanins, calculated as cyanidin‐3‐glucoside, were measured by a pH differential method using two buffer systems: potassium chloride buffer, pH 1.0 (0.025 m); and sodium acetate buffer, pH 4.5 (0.4 m; Benvenuti *et al*., 2004; Cheng and Breen, 1991). The absorbance of the solutions was measured with a UV‐4802 spectrophotometer (UNICO, Shanghai, China) at 510 and 700 nm in buffers at pH 1.0 and 4.5. The total anthocyanins content was calculated using A = [(A_510_ − A_700_)_pH 1.0_ − (A_510_ − A_700_)_pH 4.5_] with a molar extinction coefficient of cyanidin‐3‐glucoside of 26 900 and a molecular weight of 449.2. The results are expressed as mg cyanidin‐3‐glucoside equivalents per 100 g fresh weight (FW).

### Genomic DNA extraction, PCR amplification and sequencing

Total genomic DNA was extracted from 250 mg fresh leaf material with an EZ Spin Column Genomic DNA Isolation kit (Biomega Inc., Foster City, CA). Pairs of primers were synthesized by Shanghai Sangon (Shanghai, China) according to the reported sequences (Table S3). The amplification reactions were conducted in a PCR thermocycler (Techne TC312, Stone, UK) in a 20‐μL volume containing 2 μL 10× buffer, 2 μL Mg^2+^ (25 mmol), 1 μL dNTPs (10 mmol), 1 μL each primer (10 pmol), 2.5 U Taq polymerase (Sangon) and 3 μL genomic DNA (100 ng). The cycling conditions were as follows: 1 cycle at 94 °C for 4 min, 35 cycles at 94 °C for 30 s, 55 °C for 30 s and 72 °C for 2 min, followed by a final cycle at 72 °C for 10 min. The PCR products were separated on 1% (w/v) agarose gels, stained with ethidium bromide (EtBr) and visualized with a gel imaging system. The PCR fragments were purified using an EZ‐10 Spin Column DNA gel extraction kit (Bio Basic Inc., Markham, ON, Canada). The PCR products were cloned into pGEM‐T Easy (Promega, Madison, WI), cloned into the *Escherichia coli* strain XL‐Blue and sequenced.

### Sequence alignment and phylogenetic analyses

Amino acid sequence alignments of PavMYB10.1 proteins were performed using ClustalW (Chenna *et al*., 2003). The phylogenetic analysis of PavMYB10.1 was based on deduced amino acid sequences. Other MYB TF sequences were obtained from GenBank. A phylogenetic tree was generated with MEGA version 5.0 using the neighbour‐joining (NJ) method with 1000 bootstrap replicates (Tamura *et al*., 2011).

### Total RNA extraction and cDNA synthesis

Total RNA was extracted using an RNA Isolation kit (Waryong Co. Ltd., Beijing, China), according to the manufacturer's instructions. First‐strand cDNA was synthesized with a RevertAid First‐Strand cDNA synthesis kit (Thermo Scientific, Waltham, MA) according to the manufacturer's instructions. The PCR products for *PavMYB10.1* and *PavMYB10.2* were cloned into pGEM‐T Easy (Promega), cloned into the *E. coli* strain XL‐Blue and sequenced.

### Transcriptional analysis during fruit development

Total RNA was extracted from fruit at each stage of development (1–8 weeks). The first‐strand cDNA products were used as templates for PCR with specific primers (Table S3). *Actin* was used as an internal control. The PCR products were separated on 1% (w/v) agarose gels, stained with EtBr and visualized with a gel imaging system. To investigate the transcriptional patterns of various structural genes in the anthocyanin synthesis pathway at different stages of fruit development, qRT‐PCR analyses were performed with SYBR^®^ Premix Ex Taq II (Bio‐Rad, Hercules, CA) with a Bio‐Rad CF96 Real‐Time PCR Detection System. The specific primers for the structural genes are shown in Table S3. The reaction mixture (10 μL total volume) contained 5 μL SYBR Premix (2×), 1.0 μL forward primer (10 μm), 1.0 μL reverse primer (10 μm), 1 μL cDNA template (20 ng) and 2.0 μL ddH_2_O. The cycling conditions were as follows: denaturation at 95 °C for 30 s, followed by 40 cycles of 95 °C for 55 s and 60°C for 30 s. Each reaction was performed in biological triplicate. Data were analysed using the $2^{-\Delta \Delta C_{T}}$ method (Livak and Schmittgen, 2001). The transcript levels of specific genes were normalized to that of *Actin*.

### Subcellular localization of PavMYB10.1 protein

The *PavMYB10.1* ORF without a stop codon was obtained by RT‐PCR using *PavMYB10.1* primers containing digestion sites (Table S3). The PCR products were digested and cloned into the pEZS‐NL‐GFP vector. Then, pEZS‐NL‐35S:PavMYB10.1‐GFP and the control vector pEZS‐NL‐35S:GFP were introduced into onion epidermal cells by particle bombardment. The onion epidermal cells were pre‐incubated on MS organic salt plates for 24 h, and the subcellular fused protein was detected by confocal microscopy (Nikon A1R, Tokyo, Japan).

### Chromatin immunoprecipitation assay

An antibody to PavMYB10.1 was generated in a New Zealand rabbit. Initially, 200 μg purified PavMYB10.1 protein was injected into a rabbit after being mixed with Freund's complete adjuvant. Then, 8–10 booster injections were given at 10‐day intervals, and the antiserum was collected 10 days after the last injection. Purification of rabbit IgG was performed according to Pan's method (Pan *et al*., 2005). The ChIP assay was performed as described by Bowler's method (Bowler *et al*., 2004) using a Pierce™ Agarose ChIP kit (product No. 26156; Thermo Scientific) in the dark‐red ‘Lapins’ fruit. The PCR mixture (25 μL total volumes) contained the templates including 0.5 μL 10% input DNA, purified DNA by immunoprecipitated and negative DNA and primers designed from sequences in the promoter regions of *PavPAL*,* PavANS* and *PavUFGT* (Table S3). The PCR cycling conditions were as follows: 2 min predenaturation at 94 °C, followed by 35 cycles of 95 °C for 10 s, 53 °C for 30 s and 68 °C for 45 s, and a 7‐min final elongation step at 68 °C. The PCR products were separated on 1% (w/v) agarose gels, stained with EtBr and visualized with a gel imaging system.

### Yeast two‐hybrid assay

The full‐length coding sequences of *PavMYB10.1a*,* PavMYB10.1b*,* PavbHLH*,* PavWD40*, the N‐terminal sequence (1–342 bp) of *PavMYB10.1a* and the C‐terminus sequence (343–672 bp) of *PavMYB1a* were individually cloned into pGADT7 (Clontech, Palo Alto, CA) to produce fusion proteins with the GAL4 activation domain. *PavbHLH* and *PavWD40* were identified by referring to orthologous genes of *MdbHLH3* and *MdWD40* (An *et al*., 2012; Brueggemann *et al*., 2010). The full‐length coding sequences of *PavbHLH* and *PavWD40* were separately introduced into pGBKT7 to form recombinants with the GAL4 DNA‐binding domain. Various combinations of BD and AD vectors were cotransformed into the yeast strain AH109 and grown on SD/‐Leu‐Trp medium at 30 °C for 3–4 days. The clones were subsequently grown on SD/‐Ade‐His‐Leu‐Trp medium at 30 °C for 7 days to test interactions between pairs of proteins.

### Statistical analyses

The qRT‐PCR data were analysed by SPSS 16.0 (SPSS Inc., Chicago, IL) and SigmaPlot 10.0 (Systat Software Inc., San Jose, CA). Bivariate correlation analysis was used to test correlations between anthocyanin content and relative transcript levels of *PavMYB10.1* and between relative transcript levels of *PavMYB10.1* and those of other structural genes in ‘Big Dragon’, ‘Rainier’ and ‘Lapins’.

## Supporting information


**Figure S1** Ripe fruit skin and flesh colour of ‘Big Dragon’, ‘Rainier’, and ‘Lapins’.
**Figure S2** Southern blot of *PavMYB10.1* in three varieties ‘Big Dragon’, ‘Rainier’, and ‘Lapins’.
**Figure S3** Genomic DNA sequence alignment of *PavMYB10.1a* and *PavMYB10.1b* between the dark red variety ‘Lapins’ and the blush ‘Rainier’.
**Figure S4** Genomic DNA sequence alignment of *PavMYB10.2a* and *PavMYB10.2b*.
**Figure S5** Protein sequence alignment of PavMYB10.1, PavMYB10.2, and other genes from different species.
**Figure S6** Alignment cDNA of *PavMYB10.1* transcript levels in sweet cherry ‘Lapins’ and ‘Rainier’.
**Figure S7** Alignment cDNA of *PavMYB10.2a* and *PavMYB10.2b* gene.
**Figure S8** Cellular localization of *PavMYB10.1* in onion epidermal cells.
**Figure S9** Interaction of PavMYB10.1 with PavbHLH and PavWD40.
**Table S1** Correlations between anthocyanin content and relative expressions of *PavMYB10.1* and structural genes in ‘Big Dragon’, ‘Rainier’, and ‘Lapins’.
**Table S2** Correlations between relative expressions of *PavMYB10.1* and relative expressions of structural genes in ‘Big Dragon’, ‘Rainier’, and ‘Lapins’.
**Table S3** Sequences of oligonucleotide primers were used in this work.
