# Supplementary material for: The R2R3 MYB transcription factor PavMYB10.1 involves in anthocyanin biosynthesis and determines fruit skin colour in sweet cherry ( Prunus avium L.)
Source: Plant Biotechnol J. 2016 May 19;14(11):2120–33. doi: 10.1111/pbi.12568 (PMC5095807; doi:10.1111/pbi.12568)
Supplement: Supplementary file 1 — Figure S1 Ripe fruit skin and flesh colour of ‘Big Dragon’, ‘Rainier’, and ‘Lapins’. Figure S2 Southern blot of PavMYB10.1 in three varieties ‘Big Dragon’, ‘Rainier’, and ‘Lapins’. Figure S3 Genomic DNA sequence alignment of PavMYB10.1a and PavMYB10.1b between the dark red variety ‘Lapins’ and the blush ‘Rainier’. Figure S4 Genomic DNA sequence alignment of PavMYB10.2a and PavMYB10.2b. Figure S5 Protein sequence alignment of PavMYB10.1, PavMYB10.2, and other genes from different species. Figure S6 Alignment cDNA of PavMYB10.1 transcript levels in sweet cherry ‘Lapins’ and ‘Rainier’. Figure S7 Alignment cDNA of PavMYB10.2a and PavMYB10.2b gene. Figure S8 Cellular localization of PavMYB10.1 in onion epidermal cells. Figure S9 Interaction of PavMYB10.1 with PavbHLH and PavWD40. Table S1 Correlations between anthocyanin content and relative expressions of PavMYB10.1 and structural genes in ‘Big Dragon’, ‘Rainier’, and ‘Lapins’. Table S2 Correlations between relative expressions of PavMYB10.1 and relative expressions of structural genes in ‘Big Dragon’, ‘Rainier’, and ‘Lapins’. Table S3 Sequences of oligonucleotide primers were used in this work. [file PBI-14-2120-s001.pdf]

***Plant Biotechnology Journal* Supporting Information Figures S1–S9 and Tables S1–S3**

Article title: The R2R3 MYB transcription factor *PavMYB10.1* involves in anthocyanin biosynthesis and determines fruit skin colour in sweet cherry (*Prunus avium* L.)

Authors: Jin, Wanmei; Wang, Hua; Li, Maofu; Wang, Jing; Yang, Yuan; Zhang, Xiaoming; Yan, Guohua; Zhang, Hong; Liu, Jiashen; Zhang, Kaichun

The following Supporting Information is available for this article:

Figure S1. Ripe fruit skin and flesh colour of 'Big Dragon', 'Rainier', and 'Lapins'. (a) Ripe fruit skin colour of 'Big Dragon'. (b) Ripe fruit skin colour of 'Rainier'. (c) Ripe fruit skin colour of 'Lapins'. (d) Ripe fruit flesh colour of 'Big Dragon' (e) Ripe fruit flesh colour of 'Rainier'. (f) Ripe fruit flesh colour of 'Lapins'.

Figure S2. Southern blot of *PavMYB10.1* in three varieties 'Big Dragon', 'Rainier', and 'Lapins'.

Figure S3. Genomic DNA sequence alignment of *PavMYB10.1a* and *PavMYB10.1b* gene between the dark red variety 'Lapins' and the blush 'Rainier'. Introns shown in italics; black triangle indicates position of deletion, asterisk indicates stop codon.

Figure S4. Genomic DNA sequence alignment of *PavMYB10.2a* and *PavMYB10.2b* genes. Introns shown in italics and asterisk indicates stop codon.

Figure S5. Protein sequence alignment of PavMYB10.1, PavMYB10.2, and other genes from different species. Black arrows indicate Key amino acid residue difference. Double-sided arrow indicates R2 and R3 domains.

Figure S6. Alignment cDNA of *PavMYB10.1* transcript levels in sweet cherry 'Lapins' and 'Rainier'. Coding sequence alignment of *PavMYB10.1a* and *PavMYB10.1b* between dark-red variety 'Lapins' and blush variety 'Rainier'. Upwards arrow indicates position of deletion, asterisk marks stop codon.

Figure S7. Alignment cDNA of *PavMYB10.2a* and *PavMYB10.2b* gene. Coding sequence alignment of *PavMYB10.2a* and *PavMYB10.2b* between dark-red variety 'Lapins' and blush variety 'Rainier'. asterisk marks stop codon.

Figure S8. Cellular localization of *PavMYB10.1* in onion epidermal cells. Onion bulb cells were bombarded with gold particles coated with plasmids. (a) The combination of cell morphology in dark field for green fluorescence and bright light for pEZS-NL-GFP. (b) Cell morphology in dark field for green fluorescence for pEZS-NL-GFP. (c) Cell morphology in bright light for pEZS-NL-GFP. (d) The combination of cell morphology in dark field for green fluorescence and bright light for pEZS-NL-PavMYB10.1-GFP. (e) Cell morphology in dark field for green fluorescence for pEZS-NL-PavMYB10.1-GFP. (f) Cell morphology in bright light for pEZS-NL-PavMYB10.1-GFP.

Figure S9 Interaction of PavMYB10.1 with PavbHLH and PavWD40. (a) Schematics of *PavMYB10.1a* and *PavMYB10.1b* constructs used in yeast two-hybrid assays. Solid circle marks stop codon. (b) Yeast two-hybrid assays of PavMYB10.1a and PavMYB10.1b with PavbHLH and PavWD40. Indicated combinations of bait (BD fusion) and prey (AD fusion) constructs were introduced into yeast reporter strain AH109. Transformants were streaked on selective medium (SD/-Leu-Trp) and then

the single clone on medium (SD/-Leu-Trp) were inoculated on selective medium (SD/-Ade-His-Leu-Trp). Empty vectors pGBKT7 and pGADT7 were negative controls. Plates were photographed after incubation at 30℃ for 7 days.

Table S1 Correlations between anthoyanin content and relative expressions of *PavMYB10.1* and structural genes in 'Big Dragon', 'Rainier', and 'Lapins'.

Table S2 Correlations between relative expressions of *PavMYB10.1* and relative expressions of structural genes in 'Big Dragon', 'Rainier', and 'Lapins'.

Table S3 Sequences of oligonucleotide primers used in this work (F, forward; R, reverse).

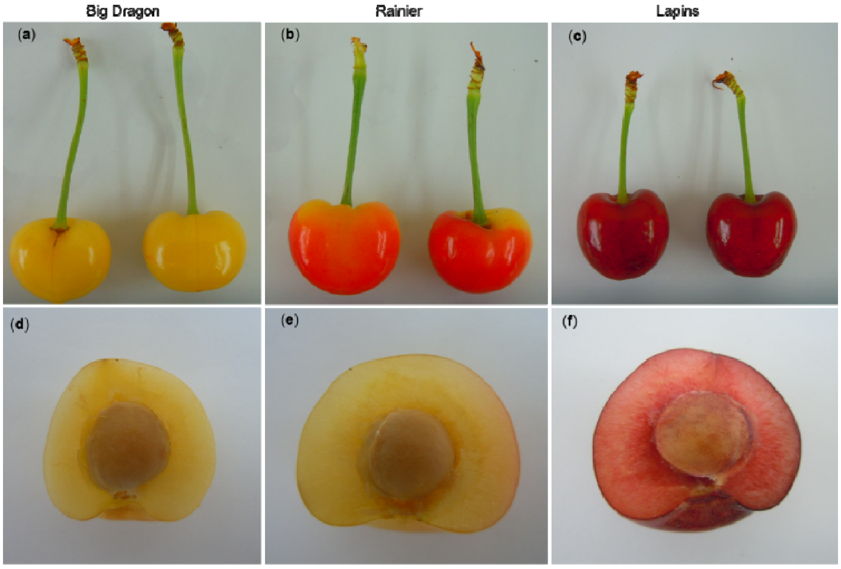

Figure S1 Ripe fruit skin and flesh colours of ‘Big Dragon’, ‘Rainier’, and ‘Lapins’. (a) Ripe fruit skin colour of ‘Big Dragon’. (b) Ripe fruit skin colour of ‘Rainier’. (c) Ripe fruit skin colour of ‘Lapins’. (d) Ripe fruit flesh colour of ‘Big Dragon’. (e) Ripe fruit flesh colour of ‘Rainier’. (f) Ripe fruit flesh colour of ‘Lapins’.

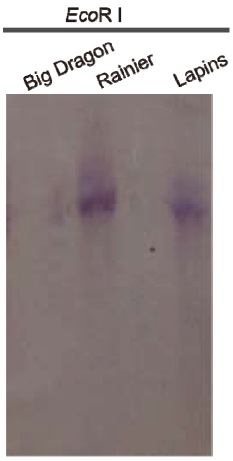

Figure S2 Southern blot of *PavMYB10.1* in three varieties ‘Big Dragon’, ‘Rainier’, and ‘Lapins’.

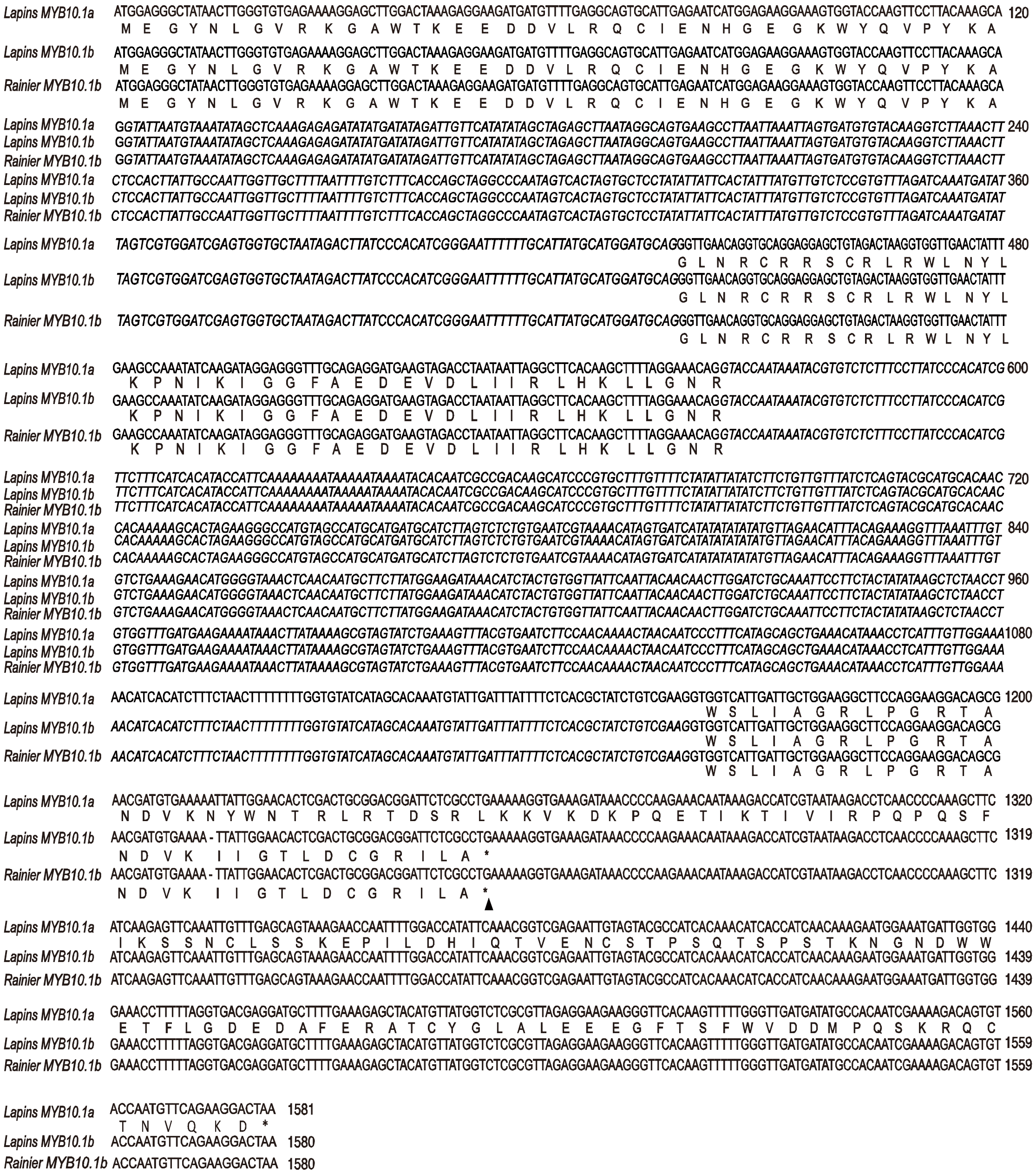

Figure S3 Genomic DNA sequence alignment of *PavMYB10.1a* and *PavMYB10.1b* between the dark red variety 'Lapins' and the blush 'Rainier'. Intron shows in italics; black triangle indicates position of deletion and asterisk indicates stop codon.

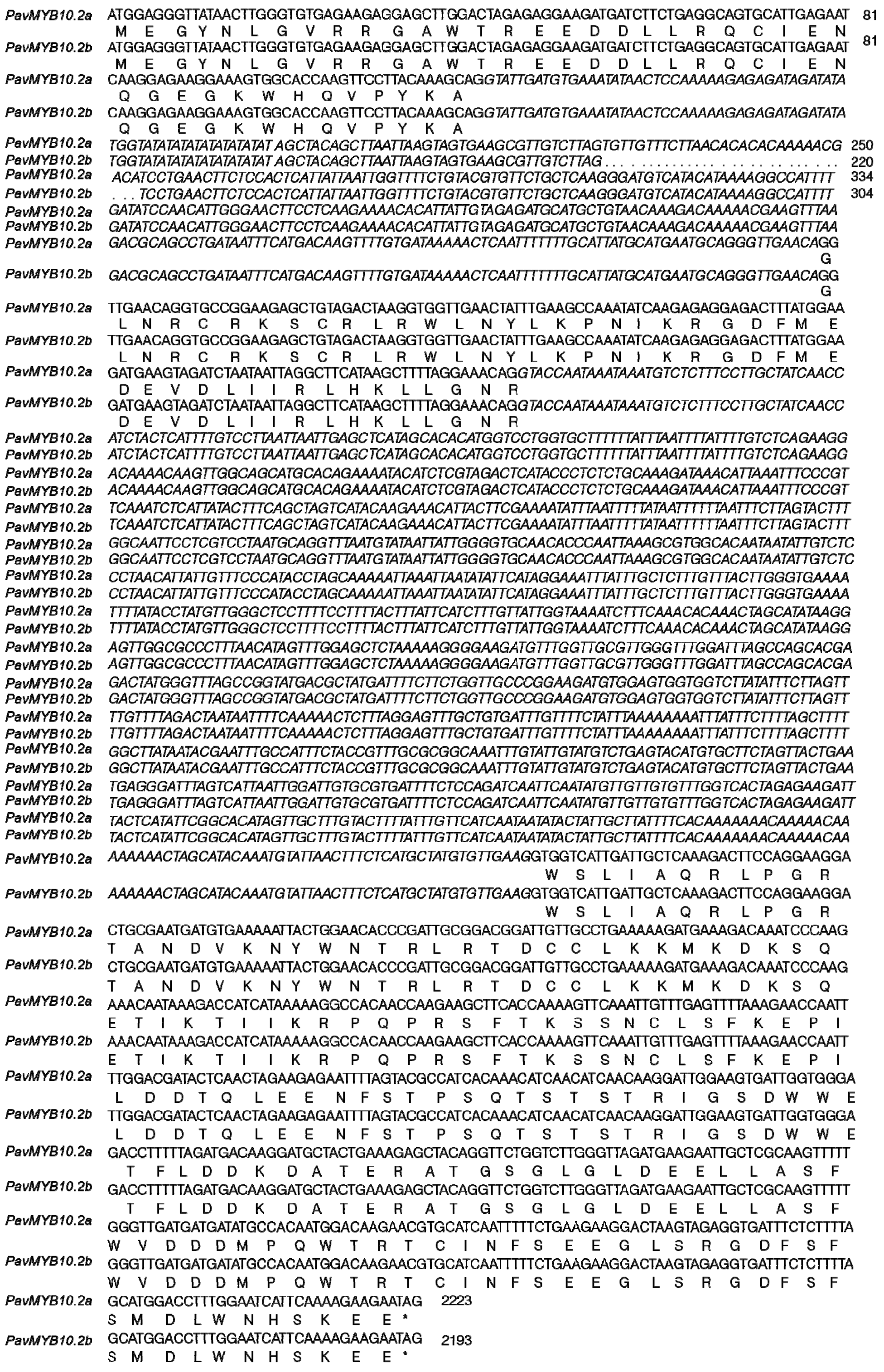

Figure S4 Genomic DNA sequence alignment of *PavMYB10.2a* and *PavMYB10.2b*.

Intron shows in italics; asterisk indicates stop codon.

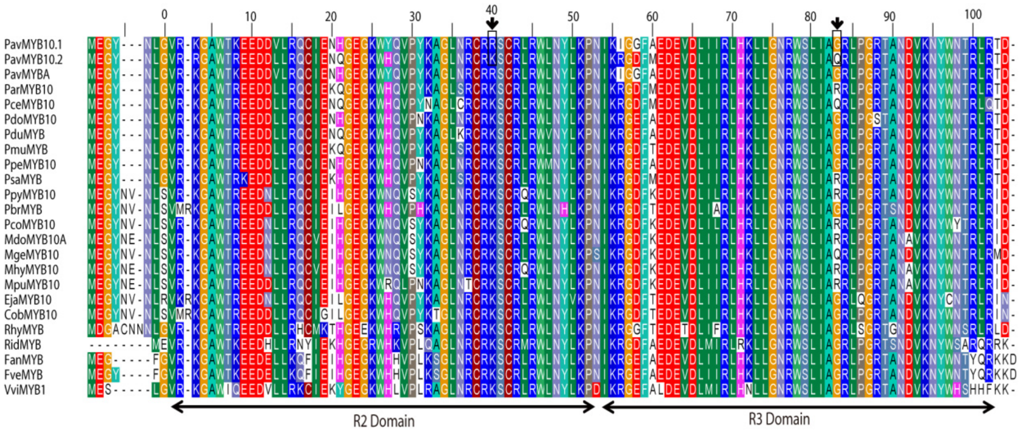

Figure S5 Protein sequence alignment of *PavMYB10.1, PavMYB10.2,* and other genes from different species. Black arrow indicates key amino acid residue difference. Double-sided arrow indicates R2 and R3 domains.

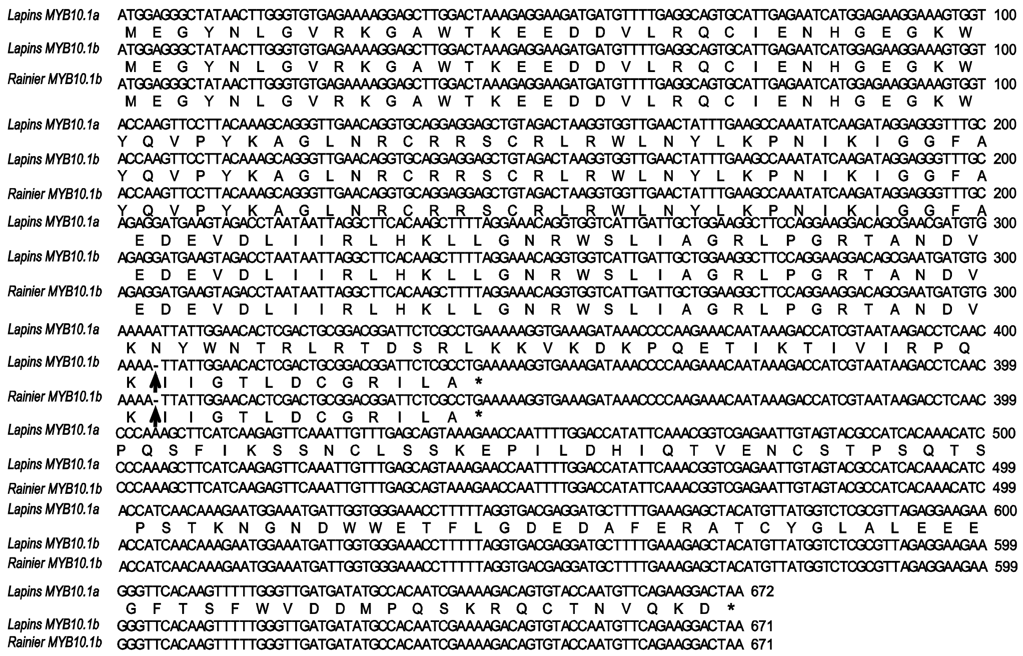

Figure S6 Alignment cDNA of *PavMYB10.1* in sweet cherry ‘Lapins’ and ‘Rainier’. Coding sequence alignment of *PavMYB10.1a* and *PavMYB10.1b* between dark-red variety ‘Lapins’ and blush variety ‘Rainier’. Upwards arrow indicates position of deletion, asterisk marks stop codon.

```
PavMYB10.2a ATGGAGGGTTATAACTTGGGTGTGAGAAGAGGAGCTTGGACTAGAGAGGAAGATGATCTTCTGAGGCAGTGCATTGAGAA 80
            M  E  G  Y  N  L  G  V  R  R  G  A  W  T  R  E  E  D  D  L  L  R  Q  C  I  E  N
PavMYB10.2b ATGGAGGGTTATAACTTGGGTGTGAGAAGAGGAGCTTGGACTAGAGAGGAAGATGATCTTCTGAGGCAGTGCATTGAGAA 80
            M  E  G  Y  N  L  G  V  R  R  G  A  W  T  R  E  E  D  D  L  L  R  Q  C  I  E  N

PavMYB10.2a TCAAGGAGAAGGAAAGTGGCACCAAGTTCCTTACAAAGCAGGGTTGAACAGGTGCCGGAAGAGCTGTAGACTAAGGTGGT 160
             Q  G  E  G  K  W  H  Q  V  P  Y  K  A  G  L  N  R  C  R  K  S  C  R  L  R  W
PavMYB10.2b TCAAGGAGAAGGAAAGTGGCACCAAGTTCCTTACAAAGCAGGGTTGAACAGGTGCCGGAAGAGCTGTAGACTAAGGTGGT 160
             Q  G  E  G  K  W  H  Q  V  P  Y  K  A  G  L  N  R  C  R  K  S  C  R  L  R  W

PavMYB10.2a TGAACTATTTGAAGCCAAATATCAAGAGAGGAGACTTTATGGAAGATGAAGTAGATCTAATAATTAGGCTTCATAAGCTT 240
            L  N  Y  L  K  P  N  I  K  R  G  D  F  M  E  D  E  V  D  L  I  I  R  L  H  K  L
PavMYB10.2b TGAACTATTTGAAGCCAAATATCAAGAGAGGAGACTTTATGGAAGATGAAGTAGATCTAATAATTAGGCTTCATAAGCTT 240
            L  N  Y  L  K  P  N  I  K  R  G  D  F  M  E  D  E  V  D  L  I  I  R  L  H  K  L

PavMYB10.2a TTAGGAAACAGGTGGTCATTGATTGCTCAAAGACTTCCAGGAAGGACTGCGAATGATGTGAAAAATTACTGGAACACCCG 320
             L  G  N  R  W  S  L  I  A  Q  R  L  P  G  R  T  A  N  D  V  K  N  Y  W  N  T  R
PavMYB10.2b TTAGGAAACAGGTGGTCATTGATTGCTCAAAGACTTCCAGGAAGGACTGCGAATGATGTGAAAAATTACTGGAACACCCG 320
             L  G  N  R  W  S  L  I  A  Q  R  L  P  G  R  T  A  N  D  V  K  N  Y  W  N  T  R

PavMYB10.2a ATTGCGGACGGATTGTTGCCTGAAAAAGATGAAAGACAAATCCCAAGAAACAATAAAGACCATCATAAAAAGGCCACAAC 400
              L  R  T  D  C  C  L  K  K  M  K  D  K  S  Q  E  T  I  K  T  I  I  K  R  P  Q
PavMYB10.2b ATTGCGGACGGATTGTTGCCTGAAAAAGATGAAAGACAAATCCCAAGAAACAATAAAGACCATCATAAAAAGGCCACAAC 400
              L  R  T  D  C  C  L  K  K  M  K  D  K  S  Q  E  T  I  K  T  I  I  K  R  P  Q

PavMYB10.2a CAAGAAGCTTCACCAAAAGTTCAAATTGTTTGAGTTTTAAAGAACCAATTTTGGACGATACTCAACTAGAAGAGAATTTT 480
            P  R  S  F  T  K  S  S  N  C  L  S  F  K  E  P  I  L  D  D  T  Q  L  E  E  N  F
PavMYB10.2b CAAGAAGCTTCACCAAAAGTTCAAATTGTTTGAGTTTTAAAGAACCAATTTTGGACGATACTCAACTAGAAGAGAATTTT 480
            P  R  S  F  T  K  S  S  N  C  L  S  F  K  E  P  I  L  D  D  T  Q  L  E  E  N  F

PavMYB10.2a AGTACGCCATCACAAACATCAACATCAACAAGGATTGGAAGTGATTGGTGGGAGACCTTTTTAGATGACAAGGATGCTAC 560
             S  T  P  S  Q  T  S  T  S  T  R  I  G  S  D  W  W  E  T  F  L  D  D  K  D  A  T
PavMYB10.2b AGTACGCCATCACAAACATCAACATCAACAAGGATTGGAAGTGATTGGTGGGAGACCTTTTTAGATGACAAGGATGCTAC 560
             S  T  P  S  Q  T  S  T  S  T  R  I  G  S  D  W  W  E  T  F  L  D  D  K  D  A  T

PavMYB10.2a TGAAAGAGCTACAGGTTCTGGTCTTGGGTTAGATGAAGAATTGCTCGCAAGTTTTTGGGTTGATGATGATATGCCACAAT 640
              E  R  A  T  G  S  G  L  G  L  D  E  E  L  L  A  S  F  W  V  D  D  D  M  P  Q
PavMYB10.2b TGAAAGAGCTACAGGTTCTGGTCTTGGGTTAGATGAAGAATTGCTCGCAAGTTTTTGGGTTGATGATGATATGCCACAAT 640
              E  R  A  T  G  S  G  L  G  L  D  E  E  L  L  A  S  F  W  V  D  D  D  M  P  Q

PavMYB10.2a GGACAAGAACGTGCATCAATTTTTCTGAAGAAGGACTAAGTAGAGGTGATTTCTCTTTTAGCATGGACCTTTGGAATCAT 720
            W  T  R  T  C  I  N  F  S  E  E  G  L  S  R  G  D  F  S  F  S  M  D  L  W  N  H
PavMYB10.2b GGACAAGAACGTGCATCAATTTTTCTGAAGAAGGACTAAGTAGAGGTGATTTCTCTTTTAGCATGGACCTTTGGAATCAT 720
            W  T  R  T  C  I  N  F  S  E  E  G  L  S  R  G  D  F  S  F  S  M  D  L  W  N  H

PavMYB10.2a TCAAAAGAAGAATAG 735
             S  K  E  E  *
PavMYB10.2b TCAAAAGAAGAATAG 735
             S  K  E  E  *
```

Figure S7 Alignment cDNA of *PavMYB10.2a* and *PavMYB10.2b*. Coding sequence alignment of *PavMYB10.2a* and *PavMYB10.2b* between dark-red variety 'Lapins' and blush variety 'Rainier'. asterisk marks stop codon.

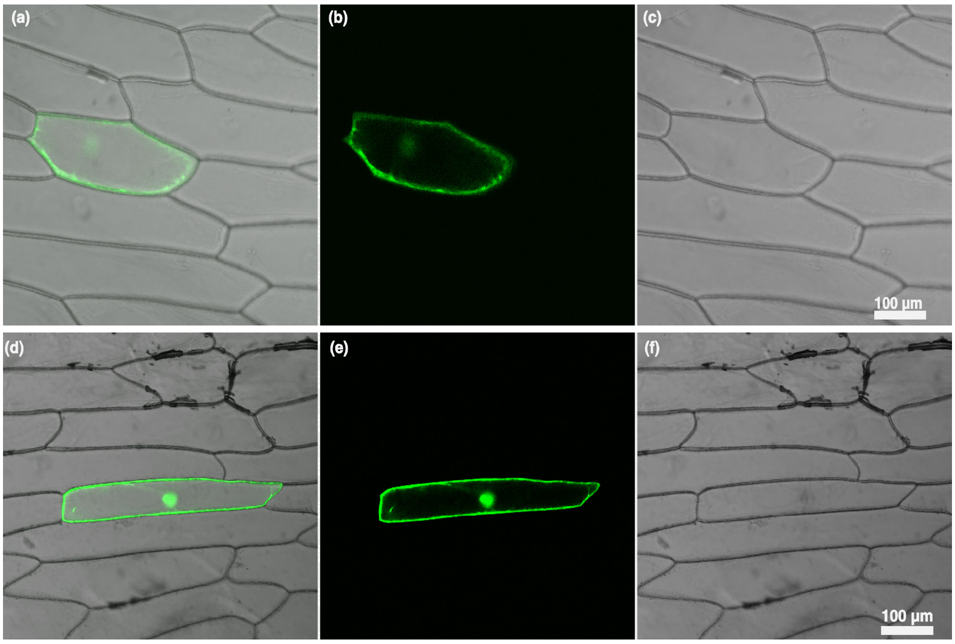

Figure S8 Cellular localization of *PavMYB10.1* in onion epidermal cells. Onion bulb cells were bombarded with gold particles coated with plasmids. (a) The combination of cell morphology in dark field for green fluorescence and bright light for pEZS-NL-GFP. (b) Cell morphology in dark field for green fluorescence for pEZS-NL-GFP. (c) Cell morphology in bright light for pEZS-NL-GFP. (d) The combination of cell morphology in dark field for green fluorescence and bright light for pEZS-NL-PavMYB10.1-GFP. (e) Cell morphology in dark field for green fluorescence for pEZS-NL-PavMYB10.1-GFP. (f) Cell morphology in bright light for pEZS-NL-PavMYB10.1-GFP.

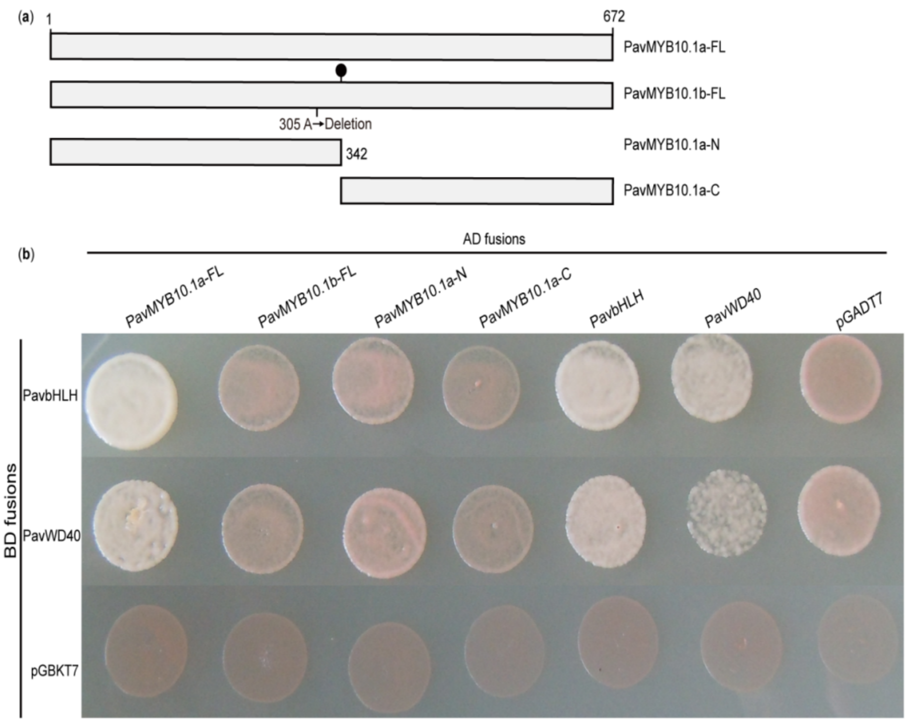

Figure S9 Interaction of PavMYB10.1 with PavbHLH and PavWD40. (a) Schematics of *PavMYB10.1a* and *PavMYB10.1b* were used in yeast two-hybrid assays. Solid circle marks stop codon. (b) Yeast two-hybrid assays of PavMYB10.1a and PavMYB10.1b with PavbHLH and PavWD40. Indicated combinations of bait (BD fusion) and prey (AD fusion) constructs were introduced into yeast reporter strain AH109. Transformants were streaked on selective medium (SD/-Leu-Trp) and then the single clone on medium (SD/-Leu-Trp) were inoculated on selective medium (SD/-Ade-His-Leu-Trp). Empty vectors pGBKT7 and pGADT7 were negative controls. Plates were photographed after incubation at 30℃ for 7 days.

Table S1 Correlations between anthoyanin content and relative expressions of *PavMYB10.1* and structural genes in 'Big Dragon', 'Rainier', and 'Lapins'.

Gene name	Big Dragon		Rainier		Lapins	
	Correlation	*p* value	Correlation	*p* value	Correlation	*p* value
PavMYB10.1	–	–	0.913**	0.002	0.854*	0.014
PavPAL	–	–	-0.416	0.306	0.288	0.488
PavC4H	–	–	-0.401	0.325	0.272	0.515
Pav4CL	–	–	-0.398	0.329	0.133	0.754
PavCHS	–	–	-0.446	0.269	0.596	0.119
PavCHI	–	–	-0.495	0.212	0.845**	0.008
PavF3H	–	–	-0.46	0.252	-0.057	0.894
PavF3'H	–	–	-0.564	0.145	0.717*	0.045
PavDFR	–	–	-0.406	0.319	0.452	0.261
PavANS	–	–	-0.122	0.773	0.845**	0.008
PavUFGT	–	–	0.780*	0.022	0.857**	0.007
PavLAR	–	–	-0.348	0.398	-0.394	0.334
PavANR	–	–	-0.447	0.267	-0.332	0.422
PavFLS	–	–	-0.34	0.41	-0.13	0.758

* Correlation is significant at the 0.05 level (2-tailed). ** Correlation is significant at the 0.01 level (2-tailed).

Table S2 Correlations between relative expression of *PavMYB10.1* and that of structural genes in 'Big Dragon', 'Rainier', and 'Lapins'.

Genes	Big Dragon		Rainier		Lapins	
	Correlation	*p* value	Correlation	*p* value	Correlation	*p* value
PavPAL	–	–	-0.364	0.376	0.785*	0.021
PavC4H	–	–	-0.311	0.454	0.241	0.565
Pav4CL	–	–	-0.298	0.473	0.023	0.957
PavCHS	–	–	-0.35	0.396	0.271	0.517
PavCHI	–	–	-0.413	0.31	0.458	0.254
PavF3H	–	–	-0.366	0.373	-0.185	0.661
PavF3'H	–	–	-0.494	0.214	0.574	0.136
PavDFR	–	–	-0.316	0.445	0.29	0.486
PavANS	–	–	-0.087	0.838	0.902**	0.002
PavUFGT	–	–	0.956**	0	0.861**	0.006
PavLAR	–	–	-0.232	0.58	-0.038	0.928
PavANR	–	–	-0.35	0.395	-0.547	0.161
PavFLS	–	–	-0.226	0.591	0.446	0.269

* Correlation is significant at the 0.05 level (2-tailed). ** Correlation is significant at the 0.01 level (2-tailed).

Table S3 Sequences of oligonucleotide primers were used in this work (F, forward; R, reverse).

Purpose	Accession	Alias*	Sequence (5→3)
qRT-PCR	JF748833	*PavCHS*	F: GCTCGTGTTCTTGTTGTGTGC
			R: ACTGTCGGGAAGGATGGTTTG
	JF740091	*PavCHI*	F: TCCACCGTCAGTCAAACCAC
			R: CCTCCAAGTACACGCCAATC
	JF740092	*PavF3H*	F: CATCGTCTCCAGCCATTTACAG
			R: ATCAGCTCGTCGCTGTACTTCTG
	KF974775	*PavDFR*	F: CATCCATGCCACCAAGCCTC
			R: AAGTGAACGAACTGCCCCCG
	KF974776	*PavANS*	F: GCCTTTTTCGATCTTCCCAT
			R: CTTCTCCAGCCTCCCTTC
	KF974777	*PavUFGT*	F: ATGTCGGACCTTTCAACCTA
			R:GGCACCACCATCCCATTACT
	GU990523	*Pav4CL*	F: CCAATGCCAAGCTCATCATCA
			R: GAGAAATGCAAGCAATTCTCTGG
	GU990522	*PavC4H*	F: GAAGATCTGCGAAGAGGTCAAA
			R: GTCCTCGTTGATCTCTCCC
	GU938688	*PavANR*	F: GACCTTGTTTTCCATGTTGCC
			R: CGACACTGCAGCTGCTGATGA
	GU938685	*PavFLS*	F: TTATTACCCACCATGTCCCCG
			R: ATGTACTTAACGTCGTACC
	GU938686	*PavLAR*	F: GGCTGACATCATCAAAGCCCT
			R: ATCTGGTCCAATATTGTTG
	AF036948	*PavPAL*	F: CATAAGATTTGAGATCTTGGAA
			R: CTGAGGGTCTGGCCATCTGGT
	JQ697494	*F3'H*	F: GTGGCCTATTGTTGGGAATTTGC
			R: GCTTGAGAAGTTGGTGTCGTGGG
	KP455680	*PavMYB*	F: GGTGGTCATTGATTGCTGGA
			R: GTGATGTTTGTGATGGCGTA
	FJ560908	*PavACTIN*	F: CCAGGGCTGTGTTTCCTTCTATT
			R: ATGATCTGCGTCATCTTTTCT
Cloning	KP455680	*PavMYB10.1*	F: ATGGAGGGCTATAACTTGGGTG
			R: TTAGTCCTTCTGAACATTGGTACA
	ABX71943	*PavMYB10.2*	R:ATGGAGGGTTATAACTTGGGTG
			F:CTATTCTTCTTTTGAATGATTCCA
Yeast two-hybrid	KP126521	*PavbHLH-EcoR* I	F:GAAATTGAATTCATGGCTGCACCGCCAAGCAGCA
		PavbHLH-Pst I	R:GAAATTCTGCAGTCAGATTGGGGAATTATTTGATTA
	KP143539	*PavWD40-Nde* I	F:GAAATTCATATGATGGAGAACTCAGCACAAGAATC
		PavWD40-EcoR I	R:GAAATTGAATTCTCAAACCTTCAAAAGCTGCATC
	KP455682	*PavMYB10.1*-FL-*Nde* I	F:GAAATTCATATGATGGAGGGCTATAACTTGGGTG
		PavMYB10.1-FL-*Xho* I	R:GAAATTCTCGAGTTAGTCCTTCTGAACATTGGTACA
	KP455683	*PavMYB10.1*-N-*Xho* I	R: GAAATTCTCGAGTCAGGCGAGAATCCGTCCG

		PavMYB10.1-C-*Nde* I	F:GAAATTCATATGAAAAAGGTGAAAGATAAACCCCAAG
Subcellularly	KP455680	*PavMYB10.1*-GFP-*Xho* I	F:GAAATTCTCGAGATGGAGGGCTATAACTTGGGTG
localized		*PavMYB10.1*-GFP-*Sac* II	R:GAAATTCCGCGGGTCCTTCTGAACATTGGTACA
Chromatin	KF974776	Promoter of *PavANS*	F:TTGATAAATTGAAAGAAACAT
immunopreci-			R: CAAGCATTTTGCTAATATTTCAAA
pitation	KF974777	Promoter of *PavUFGT*	F: CGATATTTAGAGAGAGAAACAA
			R: GATTTGGATGAGATTGGATT
	AF036948	Promoter of *PavPAL*	F: ATCCACAGATGATCATACGGTC
			R: TGGGAAAATACAAAAACTGATG
SNP marker		Marker1637	F:TCACACTGATGATGACTCATACTTTGTAATTCAGTACTTG
			R:CTAATTCTCTAAACTGTTTTTGAGAATCGAATTTGCAATG
		Marker3823	F:GCTTGTGAAACTGTACTTTTGCTTGTGTGATTTGTGTGTA
			R:CTGCTGCATATCTTTGGTCAGTTTTCAATCATTATCATTT
